# A Knockout Screen of ApiAP2 Genes Reveals Networks of Interacting Transcriptional Regulators Controlling the *Plasmodium* Life Cycle

**DOI:** 10.1016/j.chom.2016.12.003

**Published:** 2017-01-11

**Authors:** Katarzyna Modrzynska, Claudia Pfander, Lia Chappell, Lu Yu, Catherine Suarez, Kirsten Dundas, Ana Rita Gomes, David Goulding, Julian C. Rayner, Jyoti Choudhary, Oliver Billker

**Affiliations:** 1 Wellcome Trust Sanger Institute, Hinxton, Cambridge CB10 1SA, UK

**Keywords:** ap2-o, ap2-o2, ap2-o3, ap2-o4, ap2-sp, ap2-sp2, ap2-sp3, ap2-l, Apetala 2, oocyst

## Abstract

A family of apicomplexa-specific proteins containing AP2 DNA-binding domains (ApiAP2s) was identified in malaria parasites. This family includes sequence-specific transcription factors that are key regulators of development. However, functions for the majority of ApiAP2 genes remain unknown. Here, a systematic knockout screen in *Plasmodium berghei* identified ten ApiAP2 genes that were essential for mosquito transmission: four were critical for the formation of infectious ookinetes, and three were required for sporogony. We describe non-essential functions for AP2-O and AP2-SP proteins in blood stages, and identify AP2-G2 as a repressor active in both asexual and sexual stages. Comparative transcriptomics across mutants and developmental stages revealed clusters of co-regulated genes with shared *cis* promoter elements, whose expression can be controlled positively or negatively by different ApiAP2 factors. We propose that stage-specific interactions between ApiAP2 proteins on partly overlapping sets of target genes generate the complex transcriptional network that controls the *Plasmodium* life cycle.

## Highlights

• Mutants in 11 of 26 apiAP2 genes reveal gene functions in mosquito transmission
• Co-expression clustering across mutants and stages reveals molecular phenotypes
• Multifunctional apiAP2 genes create complex regulatory networks in *Plasmodium*
• *Ap2-g2* is a transcriptional repressor in both asexual and sexual blood stages

## Graphical Abstract

**Figure:**
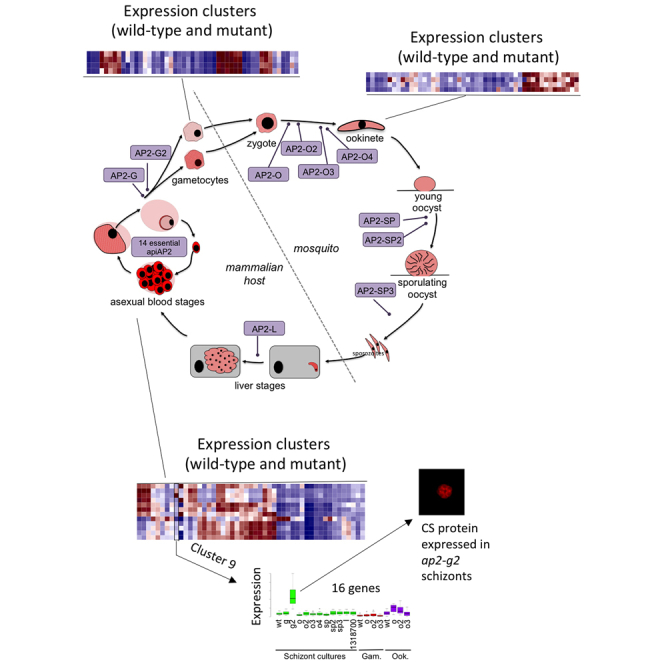

## Teaser

Modrzynska et al. investigate 25 putative transcriptional regulators of the apiAP2 family in a malaria parasite using a systematic knockout screen. Cellular and molecular phenotyping of the 11 viable mutants obtained reveals complex interactions between positive and negative regulators controlling the *Plasmodium* life cycle at different stages.

## Introduction

*Plasmodium* parasites are the causative agents of malaria and have evolved complex life cycles to alternate between a vertebrate host and an anopheline mosquito vector. Asexual parasites replicating inside red blood cells (RBCs) are responsible for all disease symptoms, but successful transmission into a new host relies on a much smaller number of sexual blood stages, the gametocytes, which remain dormant until taken up by a female mosquito. In the blood meal male (micro-) and female (macro-) gametes emerge and fertilize. After ∼20 hr, the fertilized zygote transforms into a tetraploid, motile form, the ookinete, which penetrates the midgut wall and fixes itself to the basal lamina, where it turns into an oocyst. Within 2 weeks, the cyst undergoes multiple divisions generating sporozoites, small motile forms that invade the salivary glands, from which they can be transmitted to the next mammalian host. Following one round of replication in parenchymal cells of the liver, the parasites emerge into the bloodstream, thus completing the life cycle.

Stage transitions in the life cycle are accompanied by changes in gene expression, such that the majority of *Plasmodium* genes are only induced in a narrow window (e.g., López-Barragán et al., 2011, Otto et al., 2010, Zhou et al., 2008), reflecting the marked differences in cell morphology, function, and environment. The mechanisms linking gene expression and stage transitions remain poorly understood. In higher eukaryotes, the bulk of expression control happens at the level of transcription initiation and relies on sequence-specific transcription factors, which recognize their binding sequences in a promoter, thereby influencing the recruitment of the transcription preinitiation complex and RNA polymerase. The overall positive correlation between transcription and translation dynamics (Caro et al., 2014) and between mRNA and protein levels at various stages of the life cycle (Foth et al., 2011, Tarun et al., 2008) suggests that the same is likely true for *Plasmodium*. Additionally, some promoters can drive expression of a transgene with an intensity and pattern comparable to their endogenous gene, even when removed from the native genomic context (Janse et al., 2006a, Laurentino et al., 2011, Sebastian et al., 2012). However, a variety of alternative control mechanisms have been described in *Plasmodium*, which at different life cycle stages include changes in nuclear organization, chromatin modifications (Ay et al., 2015), dynamics of RNA degradation (Shock et al., 2007), and posttranscriptional repression (Mair et al., 2006). These are also likely to contribute to both the complexity and flexibility of the gene expression network.

Intriguingly, most sequence-specific transcription factor families found in other eukaryotes seem to be absent from *Plasmodium*. Instead, an expansion of a protein family containing one or more *apetala2* (AP2) DNA-binding domains was observed across the phylum apicomplexa (Balaji et al., 2005). The AP2 domain was originally identified in plants, where it is a defining feature of a major family of AP2/ERF proteins, which control many key aspects of plant biology by acting as repressors or activators of transcription (Licausi et al., 2013). AP2 domain-containing (ApiAP2) proteins are present in all apicomplexa studied so far (Oberstaller et al., 2014), and besides one or more 60 aa AP2 DNA-binding domains, they rarely contain other known functional features. In total, 27 members of this family have been found in the human malaria parasite *Plasmodium falciparum* (although a possible 28th member of the family may be present; Oberstaller et al., 2014). In total, 26 of these have syntenic orthologs in rodent malaria species, each with its unique stage-specific expression profile (Figure S1, available online).

Recombinantly expressed *P. falciparum* AP2 domains bind specifically to a large variety of DNA sequences. Putative binding motifs were identified upstream of the majority of genes, making ApiAP2s the main candidates for generating stage-specific patterns of gene expression (Campbell et al., 2010). So far, six members of this family have been studied functionally in malaria parasites of either humans or rodents. Five were shown to play key roles in parasite progression through the life cycle and were crucial for gametocytogenesis (*ap2-g* and *ap2-g2*; Kafsack et al., 2014, Sinha et al., 2014, Yuda et al., 2015), ookinete development (*ap2-o*; Yuda et al., 2009), sporozoite formation (*ap2-sp*; Yuda et al., 2010), and liver stage maturation (*ap2-l*; Iwanaga et al., 2012). A sixth member of the family (*pfsip2*) was identified as a chromatin tethering protein, with a likely role in telomere biology in *P. falciparum*, and the overexpression of its N-terminal part containing the DNA-binding domains had no effect on gene expression (Flueck et al., 2010). However, the functions of most members of the family remain unknown.

Here we present a systematic knockout (KO) screen targeting the ApiAP2 family in the rodent malaria parasite *Plasmodium berghei*. Phenotyping of eleven viable ApiAP2 KO mutants reveals ten critical gene functions at different points in the life cycle. RNA sequencing (RNA-seq) analysis of the selected mutants at different life stages identifies changes in the transcriptomes associated with the early transmission phenotypes, and reveals clusters of functionally connected and co-regulated genes that likely share mechanisms of stage-specific control, and whose promoters we investigated for putative regulatory motifs. These data generate a deeper understanding of individual ApiAP2 proteins and how positive and negative regulators likely interact to generate complex patterns of gene expression. To test just one of the hypotheses arising from the screen, we present data supporting a general function *ap2-g2* as a repressor of transmission-specific genes, i.e., not only in gametocytes (Yuda et al., 2015) but also in asexual blood stages.

## Results

### ApiAP2 Mutants Reveal Stage-Specific Gene Functions

**Figure 1:**
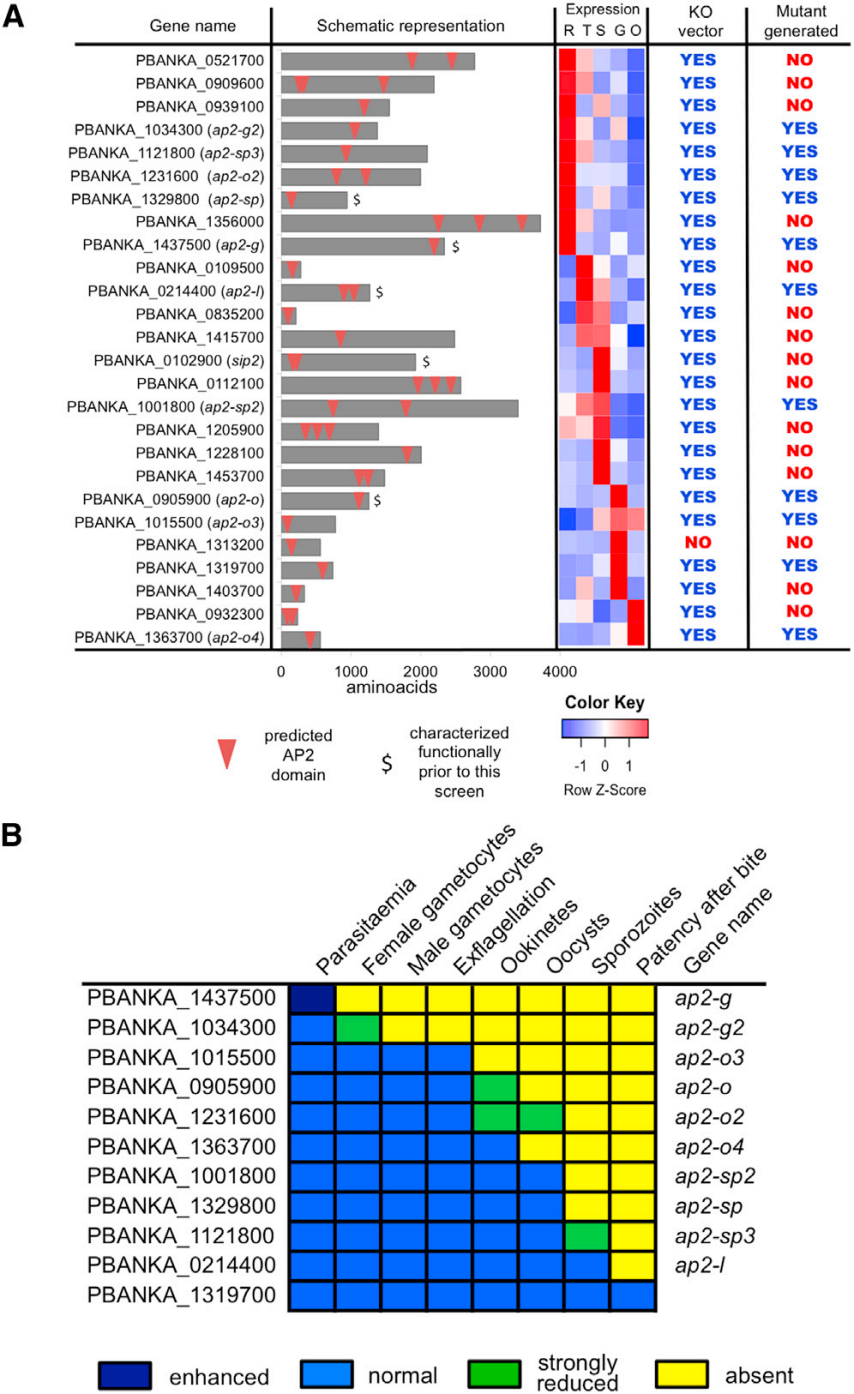
Systematic Phenotyping of ApiAP2 Gene KOs in *P. berghei* (A) Schematic representation of *P. berghei* ApiAP2 genes and KO clones obtained in this study. Expression data for ring (R), trophozoite (T), schizont (S), gametocyte (G), and ookinete (O) stage are from Otto et al. (2014). (B) Summary of a high-level phenotypic screen of eleven ApiAP2 KOs. Phenotypes were called if parasite numbers were reduced >80% or increased >20% compared to a wild-type control studied in parallel. Experiments were performed as triplicates on at least two separate occasions. Previously undescribed phenotypes were confirmed with independently created KO clones. For the complete data, see Data S1.

All 26 ApiAP2 genes of *P. berghei* possess syntenic orthologs in *P. falciparum*, *P. vivax* (main malaria species infecting humans), and *P.yoelii* (another rodent malaria model). While across the family there is much variation in gene size, numbers of predicted AP2 domains, and expression patterns, these parameters are largely conserved within orthologous groups, suggesting a high degree of functional conservation (Figures 1A and S1).

To target *P. berghei* ApiAP2 genes systematically, we succeeded in producing deletion vectors for all but one member of the family (Data S1) and transfected each of them into a reporter line constitutively expressing GFP to facilitate phenotyping. Fourteen ApiAP2 genes resisted at least four disruption attempts with up to two different vector designs, providing tentative evidence that more than half of the genes in this family are potentially essential for asexual blood stage growth in vivo. For the remaining eleven, a KO line could be generated. These included six genes that had not previously been studied in *Plasmodium* (Figures 1A and S2; Data S1).

Ten of these lines showed complete developmental lethality at some stage during sexual development or mosquito transmission (Figure 1B; Data S1). Mutants in five of these genes have been described previously. For the remainder, we followed the convention of naming genes according to the life cycle stage at which the lethal phenotype was observed (*ap2-o2*, *ap2-o3*, and *ap2-o4* for ookinete phenotypes and *ap2-sp2* and *ap2-sp3* for sporogony phenotypes). PBANKA_1319700 was the only gene whose deletion did not reveal a function, indicating the overall level of redundancy within the ApiAP2 family is low.

**Figure 2:**
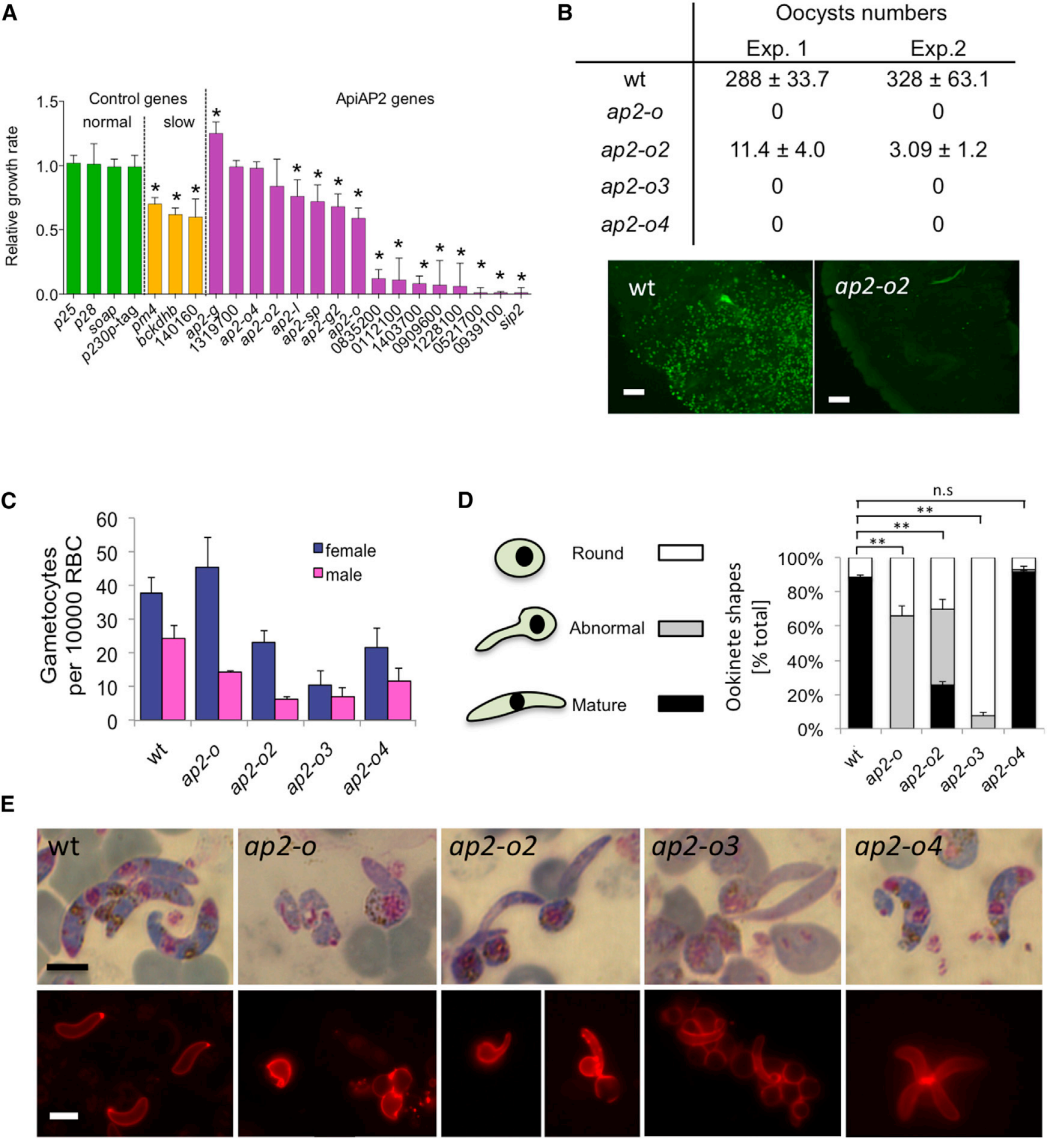
STM Analysis of ApiAP2 Family and Detailed Phenotyping of Four Mutants that Fail to Produce Infectious Ookinetes (A) Competitive fitness of 16 ApiAP2 KOs (purple) as determined by barcode counting following co-transfection of barcoded vectors. Growth rates were determined from barcode counts and are expressed relative to four mutants with wild-type growth (green). Known attenuated control mutants are shown in orange. Relative growth rates are given as the arithmetic mean of measurements taken on days 5, 6, 7, and 8 post-transfection and error bars show SDs from three transfections. Asterisk (^∗^) indicates significantly different from the pool of reference vectors with no growth effect (p < 0.05). (B) Oocysts per midgut 7 days after mosquitoes had fed on infected mice. At least 15 mosquitoes were dissected per group and arithmetic means ± SE are shown. (C) Male and female gametocytemia determined on Giemsa-stained blood films on day 3 post-infection. Error bars show SDs from three infected mice. (D) Distribution in 20 hr ookinete cultures of different parasite forms expressing the macrogamete/ookinete marker P28. At least 100 P28-positive cells were counted in each sample. Error bars show SDs from three cultures. (E) Sexual stage morphology 20 hr after gametocyte activation in culture as seen on Giemsa-stained blood films (top panels) and by live IFA using a Cy3-conjugated P28 antibody (bottom panels). Scale bar, 5 μm.

To measure effects of ApiAP2 gene deletions on asexual growth rate, we co-transfected multiple barcoded gene deletion vectors and determined the average relative growth rate of mutants during days 5–8 post-transfection by barcode counting (barseq) (Figure 2A). We used a competitive design with seven control vectors that cause known changes to parasite growth (Gomes et al., 2015). The *ap2-g* KO vector conveyed enhanced growth, as was expected, since wild-type parasites sacrifice some of their asexual reproductive potential to make gametocytes (Sinha et al., 2014). Four other mutants showed attenuated growth in asexual blood stages, including *ap2-o* and *ap2-sp*, both recognized so far only for their roles in mosquito stages.

Gametocyte number is a difficult phenotype to measure precisely in *P. berghei*, since the rate at which sexual stages are produced differs substantially between infections by the same clone and between days of the same infection (see, e.g., Sinha et al., 2014). Unsurprisingly, both parameters differed somewhat between most mutants, although they remained within the normal range in all but two mutants, *ap2-g* and *ap2-g2*, whose complete loss or severe reduction in gametocytemia has been described previously (Sinha et al., 2014). We also found no evidence for an ApiAP2 gene specifically regulating sex ratio, which in haploid *Plasmodium* blood stages is variable, but generally female biased, and not the result of chromosomally encoded sex-determining genes (Paul et al., 2000).

### ApiAP2 Genes Required for Infectious Ookinetes

Four mutants were characterized by completely or strongly reduced numbers of oocysts on the midgut epithelium of *Anopheles stephensi* mosquitoes (Figure 2B). All had gametocyte numbers and sex ratios that would not limit transmission (Figure 2C). Male gametocytes appeared mature since they readily differentiated into flagellated gametes when their development was triggered in vitro, a process termed exflagellation (Data S1), and light microscopy of activated gametocytes showed evidence of fertilization. However, zygotes of these mutants failed to varying degrees to differentiate into fully functional ookinetes in vitro (Figures 2D, 2E, S3A, and S3B). This group included *ap2-o*, in which zygotes were arrested at an intermediate stage of ookinete formation, as described previously (Yuda et al., 2009). Two additional mutants, *ap2-o2* and *ap2-o3*, also gave rise to retort-shaped intermediate ookinete forms (Figures 2D and 2E). Mutant *ap2-o3* showed the most severe defect in the ookinete group, with the majority of zygotes failing entirely to show apical complex protrusions. By comparison, *ap2-o2* produced at least some morphologically normal ookinetes in vitro and was presumably responsible for the few oocysts seen in vivo. These cysts were, however, unusually small (Figure 2B) and unable to sporulate (data not shown).

In marked contrast, *ap2-o4* mutant clones produced normal numbers of morphologically mature ookinetes in vitro (Figures 2D and 2E), but they were still entirely unable to infect mosquitoes and form oocysts in vivo (Figure 2B). Neither the gliding speed of *ap2-o4* ookinetes nor the shape of their trajectories in matrigel motility assays (Moon et al., 2009) differed from wild-type (Figures S3A and S3B), raising the possibility that their developmental defect is due to either a block at the point of midgut traversal, as has been observed with mutants in secreted traversal proteins (e.g., Dessens et al., 2001), or to reduced survival of *ap2-o4* ookinetes under in vivo conditions in the blood meal, where oxidative stress is high (Turturice et al., 2013) and availability of nutrients presumably reduced compared with optimized culture conditions (Sturm et al., 2015). None of the four ookinete stage mutants produced sporozoites in mosquito salivary glands; neither could they be transmitted back to mice by mosquito bite (Data S1).

### ApiAP2 Genes Essential for Sporogony

**Figure 3:**
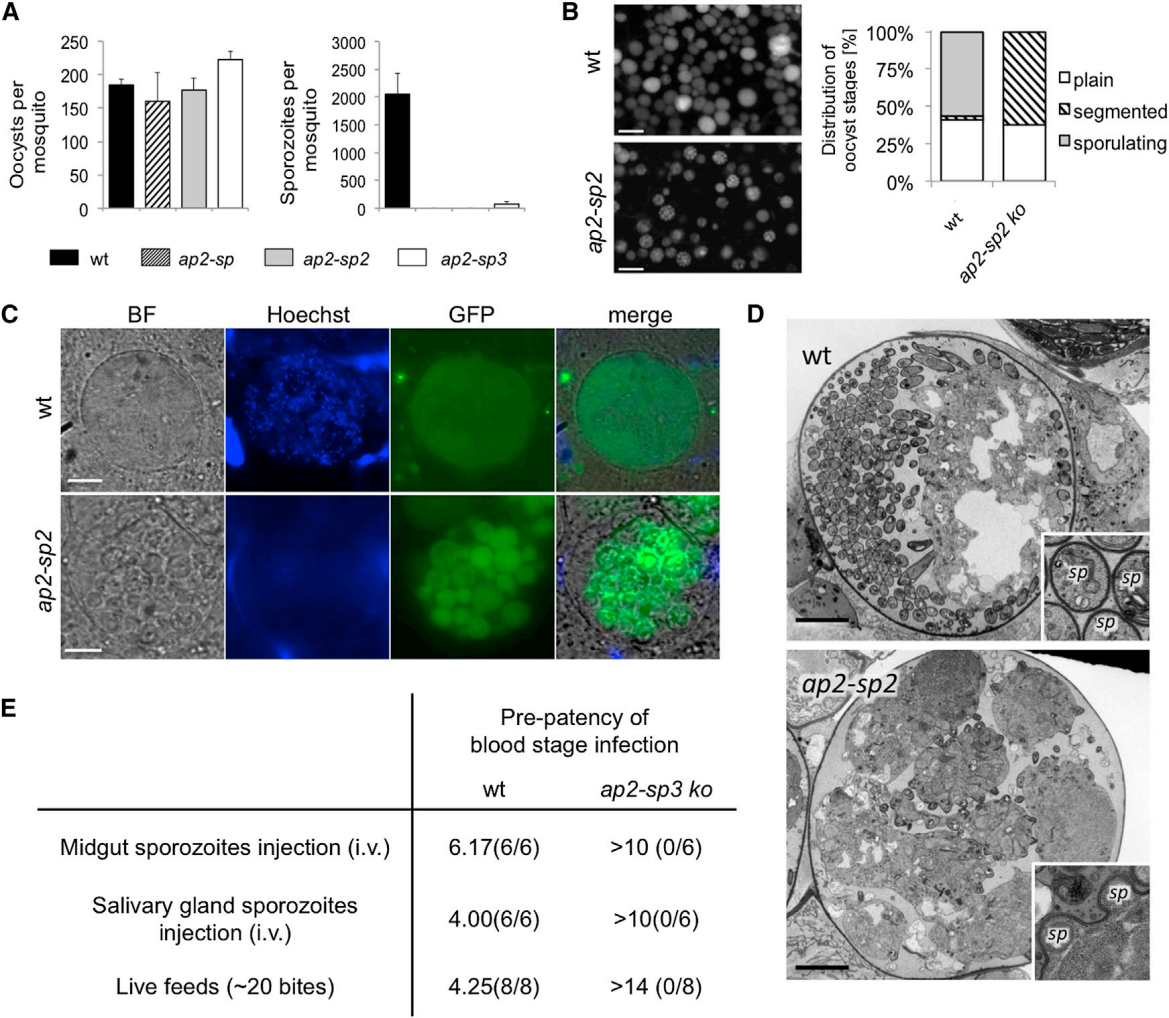
In-Depth Phenotypic Analysis of Three ApiAP2 Genes Required for Sporozoite Production (A) Midgut oocysts and salivary gland sporozoites in mosquitoes fed on infected mice. Error bars show SDs from 15 midguts (day 7 post-feeding) or 3 batches of 10 pairs of glands (day 21 post-feeding), respectively. (B) Representative fluorescence micrographs of wild-type and *ap2-sp2*-infected mosquito midguts showing different stages of sporogony as patterns of cytosolic GFP expression in oocysts on day 12 post-feeding (left; scale bar, 20 μm). The bar chart shows a quantitation of sporogonic stages averaged from ten midguts from two independent experiments. (C) Light and fluorescence micrographs of typical oocysts showing incomplete sporogony in *ap2-sp2*. Hoechst 33342 dye shows DNA. Scale bar, 5 μm. (D) TEMs showing normal sporogony in wild-type oocysts (upper panel) and incomplete budding of *ap2-sp2* sporozoites from sporogonic islands (lower panel). Scale bar, 5 μm. (E) Prepatency of blood stage infections in days following administration of sporozoites by intravenous injection, or by mosquito bite. Pooled data from independent experiments using two different *ap2-sp3* clones are shown. Prevalence is shown in brackets (infected mice/total).

Three mutants produced oocysts that were indistinguishable from wild-type in size and numbers, but failed to colonize the salivary glands with sporozoites (Figure 3A). The earliest phenotype occurred in *ap2-sp* oocysts, which were unable to enter sporogony, as described previously (Yuda et al., 2010; Figure S3C). In contrast, *ap2-sp2* oocysts were blocked later, at the sporoblast stage (Figures 3B and 3C). These oocysts ceased to mature at a point when sporozoites were beginning to form around the edges of cytoplasmic islands (Figure 3D) and remained arrested at this point until at least day 22 post-infection, resulting in the complete absence of salivary gland colonization (Data S1).

Mutants in *ap2-sp3* completed sporogony normally, as judged by cytosolic GFP fluorescence and transmission electron microscopy (TEM) (Figure S3D), yet sporozoites failed to appear in the salivary glands (Figure S3E). In contrast to a known egress mutant lacking the cysteine protease ECP1/SERA8 (Aly and Matuschewski, 2005), *ap2-sp3* sporozoites remained non-motile within cysts, and when released mechanically and injected into mice, were non-infectious (Figure 3E), suggesting *ap2-sp3* is required before egress to ensure full maturation of oocyst sporozoites. Interestingly, the small number of *ap2-sp3* sporozoites that reached the salivary glands still remained non-infectious when mosquitoes fed on naive C57BL/6 mice (Figure 3E). The only mutant that produced normal salivary gland infections but subsequently failed to infect mice was *ap2-l* (Data S1), consistent with its known essential role at the liver stage (Iwanaga et al., 2012).

### Gene Expression Changes in ApiAP2 Mutants

To reveal molecular functions of ApiAP2 genes, we studied the transcriptomes of all viable mutants by directional RNA-seq. *P. berghei* blood stage infections are asynchronous, and we therefore focused our analysis on schizonts synchronized in 22 hr in vitro cultures. It is important to note that schizont cultures also contained a small number of gametocytes (2%–10%). For three mutants blocked at the ookinete stage, we additionally investigated transcriptomes of gametocytes purified from peripheral blood, and of ookinete cultures harvested 20 hr after the initiation of fertilization in vitro, expecting these samples to provide insights into the molecular basis of the developmental phenotypes of these mutants.

**Figure 4:**
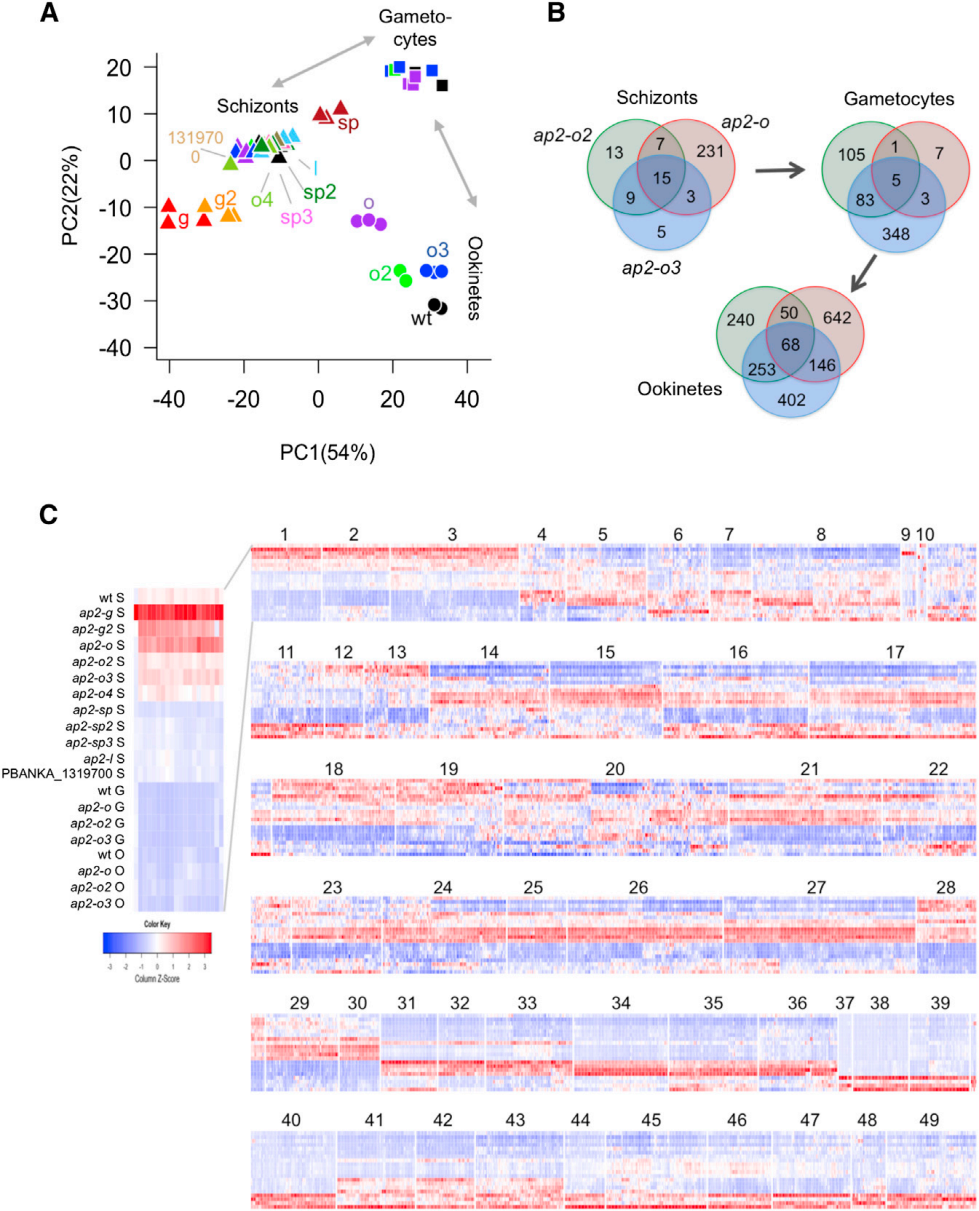
Comparative Transcriptomics of ApiAP2 KOs at Different Life Cycle Stages (A) PC analysis of all transcriptome samples. Colors indicate strains, and shapes indicate life stages. (B) Number of differentially expressed genes (absolute fold change > 2 and adjusted p value < 0.01) in three ookinete mutants at different life stages. (C) Heatmap showing the expression of 4751 *P. berghei* genes through different strain/stage combinations. The gene transcripts are ordered according to their expression patterns using Ward hierarchical clustering. The borders of 49 gene expression clusters are marked.

In a principal component (PC) analysis, transcriptomes clustered primarily by life cycle stage, and 76% of the variance was captured in the first two components (Figure 4A). Schizont samples grouped together with some spread along PC1, presumably reflecting natural variations in gametocyte numbers. Outliers were gametocyte non-producers (situated farthest away from purified gametocytes) and *ap2-sp* cultures, which were shifted toward the purified gametocytes, although gametocyte numbers in this mutant were not increased (Data S1). Gametocyte transcriptomes of the ookinete mutants grouped together, but the ookinete cultures were clearly set apart from wild-type and from each other, recapitulating when phenotypes first became apparent morphologically.

Pairwise comparisons confirmed the PC analysis, showing gametocyte genes were significantly downregulated in blood stage cultures of *ap2-g* and *ap2-g2*, but up in *ap2-sp* (Data S2). Deletion of *ap2-o* had a remarkable and unexpected effect on the transcriptome of schizont cultures, with more than 265 differentially expressed (DE) genes, potentially related to the slow growth of this mutant in mice (Figure 2A). The deregulation signature did not come from gametocytes, which had normal transcriptomes (Figure 4B). It was not enriched in known *ap2-o* targets (Kaneko et al., 2015) but was, e.g., enriched in putative RNA-binding factors (p = 0.043; Data S2). In *ap2-o2* and *ap2-o3*, the numbers of DE genes increased with developmental stage. Interestingly, in these two mutants changes in gene expression become apparent already in gametocytes, suggesting that the developmental defects observed after fertilization may have their origins in transcriptome deregulation at the earlier stage. As expected, the transcriptomes of all three ookinete mutants were significantly deregulated in the ookinete cultures (5%–15% of DE genes; Data S2; Figure 4B), but despite their similar cellular phenotypes, there was very little overlap in deregulated genes and molecular pathways (Figure 4B; Data S2).

### Co-expression Clustering Identifies Coordinated Responses to Perturbations in the ApiAP2 Network

**Figure 5:**
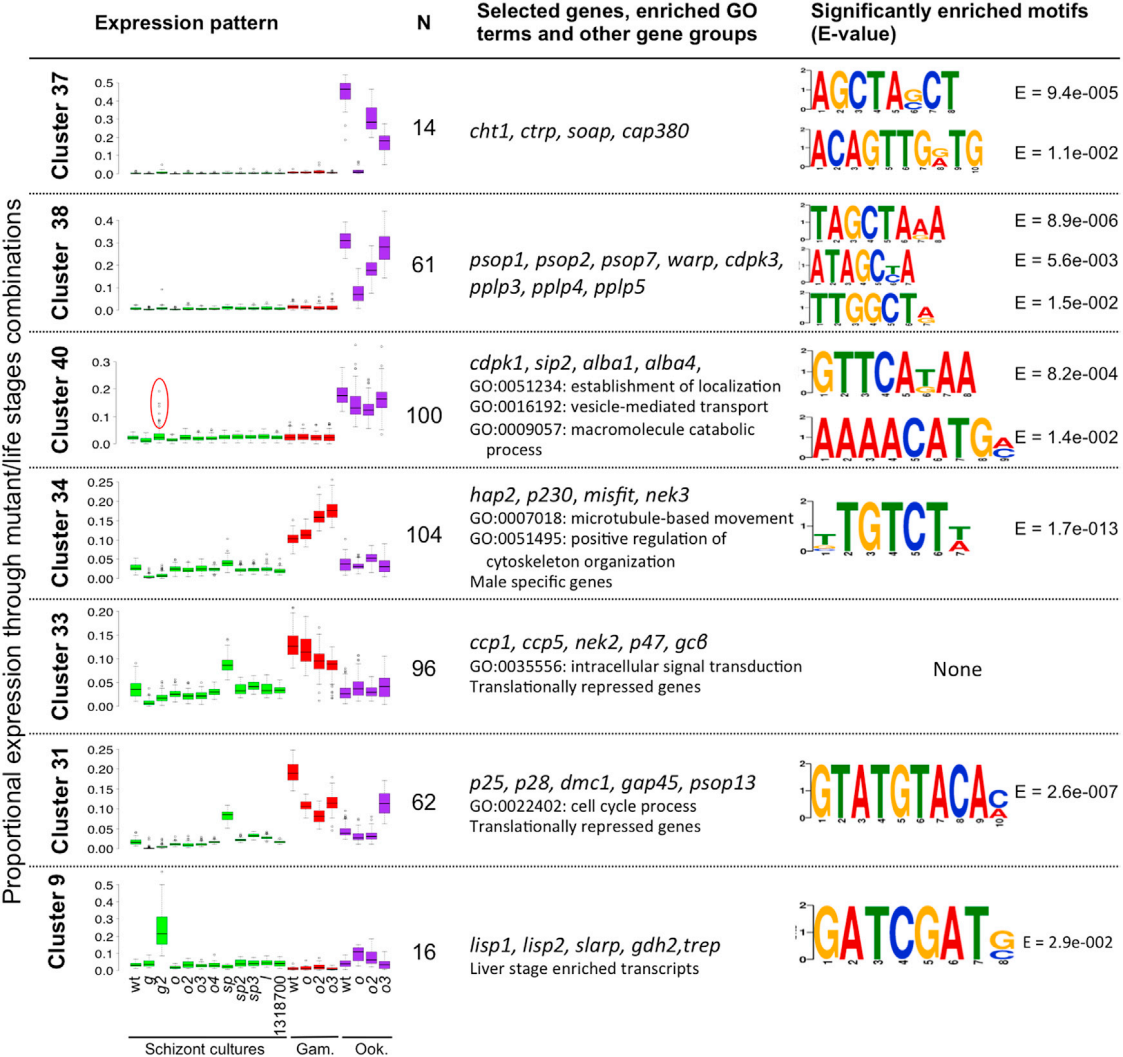
Characterization of Selected Co-expression Clusters For each stage and mutant, the average relative expression across all genes in a cluster is given. N gives the number of genes in a cluster. GO terms and functional gene groups are shown if significantly enriched (false discovery rate [FDR]-adjusted p value < 0.05). Sequence motifs shown are significantly enriched within 2 kb upstream of the start codon, as compared with all genes. See Data S3 and S4 for a comprehensive analysis of all clusters.

Co-expression clustering in *P. falciparum* has previously identified groups of functionally related genes that share conserved upstream sequences (e.g., Elemento et al., 2007, Young et al., 2008). Some of these motifs were later shown experimentally to serve as *cis*-regulatory elements recognized by specific AP2 domains (Campbell et al., 2010). Extending this concept, we wished to identify gene sets through their shared response to genetic perturbation of the ApiAP2 repertoire. Following Ward hierarchical clustering, 49 co-expression clusters were defined, each containing between 9 and 249 genes (Figure 4C; Data S3). Nearly all clusters contained functionally related genes, as evidenced by significant enrichment of gene ontology (GO) terms or metabolic pathways in 48 out of 49 clusters (Data S3 and S4). In addition, in 18 clusters we identified a total of 27 putative *cis*-regulatory elements of 8–12 bp that were significantly enriched (E value < 0.05) within 2 kb upstream of the start codon (Figure 5; Data S4).

Differential gene expression in the ookinete mutants proved a major driver for clustering. A total of 227 genes with ookinete-specific expression were distributed among four clusters (37, 38, 39, and 47) according to their degree of dependence on *ap2-o*, *ap2-o2*, and *ap2-o3* (Figure 5; Data S4). Cluster 37, for instance, contained 14 genes encoding mostly well-known ookinete microneme proteins, whose expression was entirely dependent on *ap2-o*. All four clusters were significantly enriched for variants of the known AP2-O-binding motif (E = 1.9 × 10^−4^ to 2.4 × 10^−12^) and for genes with published chromatin immunoprecipitation (ChIP) evidence (Kaneko et al., 2015) of AP2-O binding in ookinetes (p = 7.8 × 10^−7^ to 1.6 × 10^−29^). Although not all genes upregulated in ookinetes were *ap2-o* dependent (see cluster 40 in Figure 5), these data generally confirmed the central role for *ap2-o* in ookinete gene expression (Yuda et al., 2009).

By comparison, neither *ap2-o2* nor *ap2-o3* was strictly essential for any cluster of genes. A moderate downregulation of most ookinete-specific genes in the *ap2-o2* KO may only reflect the reduced number of mature ookinetes in these cultures. In contrast, *ap2-o3* clearly differentiated between groups of genes, some with well-characterized functions in ookinete gliding and invasion (compare, for instance, clusters 37 and 38 in Figure 5). Unfortunately, the known binding motif of one of the two AP2-O2 DNA-binding domains (TGACATCA) was not associated with any of the affected gene expression clusters, and the binding specificity of AP2-O3 is not known, making it impossible to predict direct targets of AP2-O2 or AP2-O3 proteins.

One clue for a function for *ap2-o3* in ookinetes is provided by cluster 31 (Figure 5), which is highly enriched (p = 4.6 × 10^−44^, 53 of 62 elements in the cluster) in genes known to be transcribed in macrogametocytes, but whose mRNAs are translationally repressed until gametocytes enter the mosquito (Mair et al., 2006). This cluster includes genes for the major ookinete surface antigens P25 and P28, but also the putative meiotic recombination protein Dmc1. For these genes, mRNA abundance usually peaks in gametocytes, but in ookinete cultures of *ap2-o3* they fail to get downregulated, which could be the result of reduced transcript turnover or a failure to repress transcription. Other genes failing to be repressed in *ap2-o3* ookinete cultures are involved in energy metabolism (cluster 45), which in the ookinete gets reorganized, and in nuclear division (cluster 31), suggesting *ap2-o3* may be required for zygotes to progress successfully beyond meiosis.

Importantly, the developmental phenotypes of *ap2-o2* or *ap2-o3* mutants at the point of ookinete formation were associated with perturbed transcriptomes already at the preceding gametocyte stage (Figure 4B). Genes upregulated in *ap2-o2* or *ap2-o3* gametocytes fell into three expression clusters (34, 35, and 36), all strongly enriched in genes encoding the male-specific gametocyte and gamete proteomes (Khan et al., 2005, Tao et al., 2014). Genes in these clusters included the male gamete fertilization factors P48/45 and HAP2, as well as putative components of the flagellar axoneme, which were all independent of *ap2-o*. Sex ratios observed on stained blood films did not support a marked shift toward male gametocytes in *ap2-o2* and *ap2-o3* mutants (Figure 2C), and these data therefore raise the possibility that dysregulation of male-specific genes translates into differences in fertility or post-fertilization development. An AGACA motif enriched in two of the male gene clusters (Figure 5; Data S4) was found previously in male genes (Young et al., 2008), but does not resemble any of the motifs known to be recognized by recombinant AP2 domains.

### A Putative Role for ap2-sp in Macrogametocyte Gene Repression

A notable feature of macrogametocyte genes in clusters 33 and 42 is their marked upregulation in schizont cultures of *ap2-sp*, which accounts for the more gametocyte-like transcriptomes of these samples (Figure 4A). In addition to its established role as direct activator of sporozoite genes in oocysts (Yuda et al., 2010), *ap2-sp* may function as a negative regulator of female-specific genes in gametocytes, or possibly even in asexual blood stages, which could explain the mild growth defect associated with the *ap2-sp* KO vector in the barseq experiment (Figure 2A). It remains to be established how *ap2-sp* could perform such additional roles, since the well-characterized binding motif targeted by its only AP2 domain is not enriched in gametocyte gene clusters (De Silva et al., 2008). Regulation of macrogametocyte genes by *ap2-sp* may be indirect, possibly involving another ApiAP2 gene. A strong candidate is PBANKA_1313200, whose expression peaks in gametocytes, where its mRNA associates with the translation repression complex until activation (Mair et al., 2006), and which is 5-fold overexpressed in *ap2-sp* blood stage cultures (Figure S4). PBANKA_1313200 has a single AP2 domain of unknown specificity, as the previous work failed to identify a putative binding motif for its *P. falciparum* ortholog, PF3D7_1449500 (Campbell et al., 2010). This is the only ApiAP2 member for which we failed to generate a targeting vector.

### AP2-G2 Is a General Repressor of Transmission Genes in Blood Stages

Disruption of *ap2-g2* results in a loss of mature gametocytes (Figure 1B) and in reduced competitive growth of asexual blood stages (Figure 2A). It was also accompanied by a marked upregulation of sporozoite and liver stage genes in blood stage cultures in two previously published datasets (Sinha et al., 2014, Yuda et al., 2015). Transcriptional phenotyping, chromatin-binding studies, and reporter assays all converged to show that in *P. berghei* gametocytes, AP2-G2 represses genes from other life cycle stages, including *lisp2* and *msp1*, by binding directly to the promoter region of its target genes (Yuda et al., 2015). The inappropriate derepression in the KO gametocytes may explain its developmental phenotype, if it causes gametocytes to degenerate before they become functionally and morphologically mature.

Consistent with this hypothesis, our current RNA-seq analysis shows sporozoite and liver stage genes repressed by *ap2-g2* strongly enriched in cluster 9, with additional deregulated genes appearing among the ookinete genes in cluster 40 (Figure 5, circle). One of the upregulated genes, however (encoding the circumsporozoite protein [CS], the major surface protein of sporozoites), was so abundant in *ap2-g2* schizont cultures that we suspected transcriptional dysregulation was not limited to the small number of “contaminating” gametocytes but more likely extended to the schizonts that accounted for the majority of parasites in the cultures. Importantly, this could help explain the reduced asexual growth rate of the *ap2-g2* mutant in vivo.

**Figure 6:**
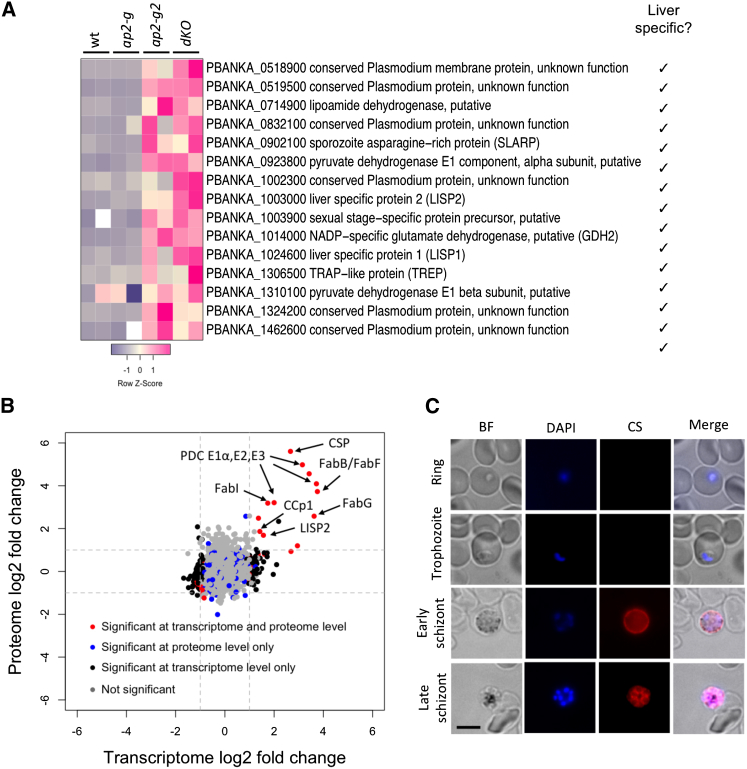
A Double Mutant with *ap2-g* Identifies *ap2-g2* as Repressor in Asexual Blood Stage (A) Heatmap showing expression of cluster 9 genes in blood stage cultures. Row-scaled reads per kilobase of transcript per million mapped reads (RPKM) counts for two replicates are shown for each parasite line: wild-type, both single mutants, and the *ap2-g/ap2-g2* double mutant (dKO). Genes previously identified as strongly upregulated at the liver stage (Tarun et al., 2008) are marked. (B) Assessing the impact on schizont proteome and transcriptome of deleting *ap2-g2* in a gametocyte-deficient *ap2-g* mutant. Normalized transcript and protein ratios for 2,169 genes detected as both transcript and protein are plotted against each other. See Data S5 for the underlying datasets. (C) Fluorescence micrographs showing aberrant expression and localization of the sporozoite surface protein CSP in schizonts of the *ap2-g/ap2-g2* double mutant. Fixed and permeabilized infected RBCs were stained with Cy3-conjugated CSP monoclonal antibody. Schizonts from an ap2-g2 single mutant also expressed CSP, while no fluorescence was observed in *ap2-g* schizonts (Figure S6A). Scale bar, 5 μm.

To address this possibility, we generated a double mutant with *ap2-g* (Figure S5), reasoning that this would block sexual development already at the point of commitment, such that all remaining deregulation would have to originate from asexual blood stages. The double mutant lacked gametocytes, as expected, but dysregulation of cluster 9 sporozoite and liver stage genes was reproduced (Figure 6A). Furthermore, despite the absence of gametocytes in the *ap2-g* genetic background, we additionally observed upregulation of gametocyte-specific and ookinete-specific genes upon deleting *ap2-g2* (Data S5). Importantly, transcriptional derepression of these genes led to expression of the corresponding proteins in asexual cultures, as shown by a proteometric analysis (Figure 6B; Data S5). Ectopically expressed proteins in double KO schizonts included CS; the gametocyte surface protein CCP1; the liver stage exported protein, LISP2 (Orito et al., 2013); nearly the complete type II fatty acid biosynthesis pathway of the apicoplast, including three components of the pyruvate dehydrogenase complex (PDC); as well as FabI, FabG, and FabB/FabF proteins, which in wild-type parasites are essential only in liver stages (Yu et al., 2008). Double KO schizonts produced substantial amounts of CS protein, which ectopically localized to the merozoite surface (Figure 6C). CS was not expressed in the parental *ap2-g* line (Figure S6A). CS was also absent from ring and trophozoite stages of the double mutant (Figure 6C), showing that some stage-specific control was maintained, probably involving additional transcriptional regulators other than AP2-G2. Taken together, these data showed that AP2-G2, in addition to its role in the gametocyte development, is important for transcriptional repression in schizonts, but that its targets differ significantly at that stage.

## Discussion

In a systematic KO screen, we have here revealed new cellular functions for five ApiAP2 genes, doubling the number of family members known to be required for parasite transmission by mosquitoes. Additionally, 14 genes that have resisted disruption are likely to function in asexual replication and can now be investigated further using conditional knockdown approaches that have recently become available in *P. berghei* (Philip and Waters, 2015, Pino et al., 2012). Additional work will be required to define more precisely the primary targets of *ap2-o2*, *ap2-o3*, *ap2-o4*, *ap2-sp2*, and *ap2-sp3*; their DNA-binding specificities; and how these relate to the cellular phenotypes of the mutants. However, the comparative analysis of how stage-specific transcriptomes are perturbed by the disruption of different ApiAP2 genes already reveals an unexpected complexity in gene regulatory networks in *Plasmodium*.

### AP2 Genes Act Together to Create Complex Patterns of Gene Expression

In contrast to what the single-gene KO studies seemed to indicate, drastic changes in gene expression in *Plasmodium* emerge not merely from a small number of stage-specifically expressed transcriptional activators, but rather from the net effect of different DNA-binding proteins acting in concert. This is perhaps best illustrated by the role of AP2-G2 as a versatile repressor in different parasite stages. In gametocytes, for instance, repression of one set of genes by AP2-G2 presumably needs to be overcome by AP2-G during sexual differentiation, but other activating ApiAP2 proteins need to overcome repression of other genes in asexual blood stages. Other ApiAP2 proteins with pleiotropic functions include *ap2-o* and *ap2-sp*, which we here show to contribute to normal gene expression and growth in asexual blood stages, in addition to their published functions as direct transcriptional activators in ookinetes and sporozoites, respectively (Yuda et al., 2009, Yuda et al., 2010). This is reminiscent of model eukaryotes, where complex gene expression patterns are the result of hierarchical networks, within which transcription factors function in combination and sometimes synergistically (Gerstein et al., 2012, Levo and Segal, 2014). In such networks, both recognition of the binding site and the direction and magnitude of the resulting regulation depend strongly on the context provided by other binding sites (Stampfel et al., 2015), adding to the plasticity of the whole system. It appears that the *Plasmodium* transcriptome is regulated in a similar way and that ApiAP2 proteins are key factors involved in this process.

A particularly striking example of a set of transcripts that change in a large number of ApiAP2 gene KOs is provided by cluster 31 (Figure 5). Importantly, this cluster is made up mostly of mRNAs, whose increased abundance in macrogametocytes is known to rely critically on posttranscriptional stabilization (Mair et al., 2006). The relatively high abundance of these transcripts in ookinete cultures of the *ap2-o3* mutant may therefore not reflect increased expression but reduced turnover, possibly due to developmental arrest. First, this serves as a reminder that transcript abundance is an imperfect proxy for promoter activity. Second, it illustrates that RNA-seq cannot distinguish deregulation events that are due to the direct action of an ApiAP2 protein on a target gene from indirect effects. That such indirect effects must strongly impact the transcriptomes of ApiAP2 mutants is illustrated by a network representation of how disrupting individual ApiAP2 genes affects many other members of this gene family, thereby probably affecting their target genes indirectly (Figure S6B). Hypotheses generated by the current analysis will therefore need to be tested in vitro and in vivo using binding studies and reporter assays. These limitations notwithstanding, comparative phenotyping by RNA-seq has revealed an unexpected complexity of gene expression control in *Plasmodium*, which can now be investigated further to gain a deeper understanding of how the *Plasmodium* life cycle is regulated and to assess how parasites can respond to environmental conditions, such as drug treatment.

## Experimental Procedures

### Animals and Parasite Strains

Parasites were maintained and phenotyped in female Theiler’s Original (TO) outbred mice of 6–12 weeks of age. All animal procedures were conducted under a project license issued by the UK Home Office and with local ethical approval. All mutants were generated on a *P.berghei* ANKA background stably expressing a GFP/luciferase fusion protein throughout the life cycle (RMgm-29) (Franke-Fayard et al., 2008).

### KO Vector Generation and Transfections

KO vectors were designed and produced as described previously (Pfander et al., 2011) and are available as part of the *Plasmo*GEM resource (http://plasmogem.sanger.ac.uk). For transfections, 1 μg vector was released from the plasmid backbone by restriction with an *NotI* and electroporated into purified schizont-stage parasites using a standard protocol (Janse et al., 2006b). Following selection with pyrimethamine, resistant parasites were initially genotyped for the presence of the selection cassette by PCR, cloned by limiting dilution, and then genotyped in-depth by PCR and southern hybridization of separated chromosomes, as appropriate.

### Phenotyping of the Mutant Clones

For blood stage and early sexual stage phenotyping, three mice were injected intraperitoneally with 150 μLof 6 mg/mL phenylhydrazine to induce reticulocyte formation, followed by an intraperitoneal injection of 5 × 10e7 parasitized RBCs 2 days later. Asexual parasitemia and male and female gametocytemias were assessed using Giemsa-stained thin blood smears on day 3 post-infection. Exflagellation efficiency was determined in the same animals by mixing 5 μL blood from the tail vein into 500 μL exflagellation medium (RPMI 1640 containing 25 mM HEPES, 4 mM sodium bicarbonate, 5% FCS, and 100 μM XA [pH 7.4]) and counting exflagellation centers per 1,000 RBCs 12–15 min afterward in a Neubauer heamocytometer. Ookinete conversion assays were performed by adding 500 μL blood to 10 mL full ookinete medium (RPMI1640 containing 25 mM HEPES, 10% FCS, and 100 μM XA [pH 7.5]) and incubating at 19°C for 20 hr. The efficiency of conversion and morphology of the cells were assessed on Giemsa-stained thin films and by live microscopy using Cy3-conjugated 13.1 monoclonal anti-P28 antibody staining.

To investigate mosquito stages, ∼100 female *Anopheles stephensi* mosquitoes, 6–9 days old, were allowed to feed for 20 min on 2 mice that 3 days earlier had been injected with 5 × 10e7 parasitized RBCs. Seven days later, midguts of 20 mosquitoes were dissected and photographed under fluorescence illumination. The pictures were then processed using Fiji software with particle counter plug-in to quantify the number and size of oocysts. On day 14, oocysts were additionally imaged using bright field illumination to monitor sporulation within the oocysts. On day 21, salivary glands of two batches of 10 mosquitoes were dissected and homogenized, and the number of sporozoites was quantified in a hemocytometer. The capacity of sporozoites to infect a new host was assessed using the same batch of mosquitoes on day 24 post-infection by allowing all remaining mosquitoes to feed for 10 min on three naive mice that were then monitored daily for the appearance of blood stage parasites.

In all phenotyping experiments, a wild-type parasite was included alongside the mutant to control for biological and technical variability between the experiments. At least two blood stage and two mosquito stage phenotyping experiments were performed for each of the mutants. For the parasites not described previously, this was repeated with two independent KOs. See Supplemental Experimental Procedures for additional phenotyping assays.

### Transcriptome Sequencing and Analysis

Schizonts, gametocytes, and ookinetes were purified and used for RNA-seq library production as described in the Supplemental Experimental Procedures. Pools of indexed libraries were sequenced on an Illumina HiSeq2500 using 100 bp paired-end reads, and the read counts for all predicted *P. berghei* transcripts were generated. R 3.0 software with the DEseq2 package was used for PC and differential expression analyses. To cluster expression profiles, reads were first normalized for library size and genes with <50 reads across all samples were removed. For each remaining gene and experimental condition, the mean percentage of the normalized reads for all replicates was then expressed as a proportion of all reads for that gene. Clusters of co-regulated genes were identified by Ward’s hierarchical clustering with a cut-off set to 49 clusters.

The enrichment for GO terms, metabolic pathways, and stage-specific datasets was performed using Fisher’s exact test. For motif analysis, a command line version of DREME (Discriminative Regular Expression Motif Elicitation) (Bailey, 2011) was used with the predicted motif size of 4–10 bp and cut-off E value of 0.05. The 2 kb sequence upstream of all genes from the cluster was used as a test sequence and the remaining promoters in the genome as a reference dataset. Further details of data analysis are available in the Supplemental Experimental Procedures section, and the full dataset was submitted to the Gene Expression Omnibus (GEO: GSE80634).

### Proteome Analysis

Purified schizont samples were prepared as for the transcriptome analysis, total protein was extracted from parasite pallet of three replicates per clone and digested with trypsin, and samples were labeled using TMT10plex technology (Thermo Fisher). The *ap2-g* replicates served as reference samples (see Supplemental Experimental Procedures for further details).

## Author Contributions

K.M. and O.B. conceived of and designed the study, with initial input from C.P. K.M. and C.P. generated the KO vectors and parasites lines. K.M., A.R.G., K.D., and C.S. conducted the phenotyping assays. D.G. performed TEM. K.M. and L.C. generated and analyzed RNA-seq data. L.Y. and J.C. generated and analyzed mass spectroscopy data. O.B. and J.C.R. supervised the work. K.M. and O.B. wrote the manuscript with contributions from all authors.

## Accession Numbers

The Gene Expression Omnibus accession number for the RNA-seq data from the apiAP2 knockouts reported in this paper is GEO: GSE80634. The ProteomeXchange Consortium accession number for the proteome sequences reported in this paper is ProteomeXchange: PXD005180.

## Supplemental Information

Document S1. Supplemental Experimental Procedures and Figures S1–S6

Data S1. Summary of the apiAP2 KO Screen and Phenotyping of the Generated Mutants Related to Figure 1.

Data S2. Transcriptome Analysis of the apiAP2 Mutants Related to Figure 4.

Data S3. Groups of Genes Co-expressed across Different apiAP2 Mutants Related to Figures 4 and 5.

Data S4. Characterization of the 49 Co-expression Clusters Generated from ApiAP2 Expression Data Related to Figures 4 and 5.

Data S5. Transcriptome and Proteome Analysis of the Double *ap2-g/ap2-g2* KO Related to Figure 6.

Document S2. Article plus Supplemental Information
