# Supplementary material for: A Knockout Screen of ApiAP2 Genes Reveals Networks of Interacting Transcriptional Regulators Controlling the Plasmodium Life Cycle
Source: Cell Host Microbe. 2017 Jan 11;21(1):11–22. doi: 10.1016/j.chom.2016.12.003 (PMC5241200; doi:10.1016/j.chom.2016.12.003)
Supplement: Document S1. Supplemental Experimental Procedures and Figures S1–S6 [file mmc1.pdf]

Cell Host & Microbe, Volume *21*

Supplemental Information

A Knockout Screen of ApiAP2 Genes Reveals Networks of Interacting Transcriptional Regulators Controlling the *Plasmodium* Life Cycle

Katarzyna Modrzynska, Claudia Pfander, Lia Chappell, Lu Yu, Catherine Suarez, Kirsten Dundas, Ana Rita Gomes, David Goulding, Julian C. Rayner, Jyoti Choudhary, and Oliver Billker

Supplemental figures:

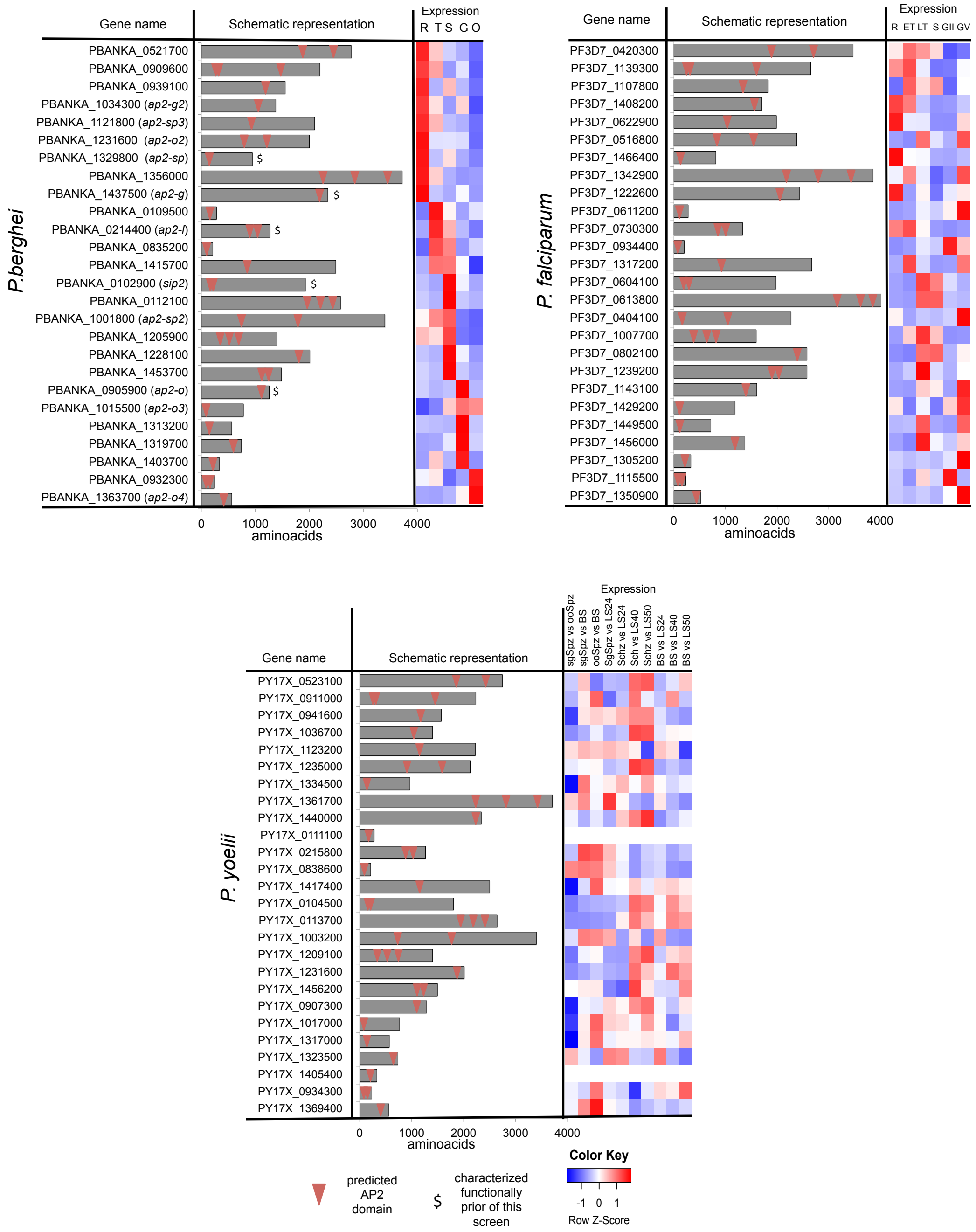

Figure S1, related to Figure 1. Comparison of gene structure and expression profiles of the members of ApiAP2 family across three different *Plasmodium* species.

Gene names and structures presented according for the latest annotation for each of the species (http://plasmodb.org). One *P.falciparum* gene with no *P.berghei/P.yoelii* ortholog has been omitted**.** RNA-seq (*Pb* and *Pf*) and microarray (*Py*) expression data extracted from Otto et al., 2014 (*Pb*),López-Barragán et al., 2011(*Pf*) and Tarun et al., 2008(*Py*). R=rings, T= trophozoites, S/Schz = schizonts, G = gametocytes, O=ookinetes, ET= early schizonts, LT= late schizonts, GII= gametocytes stage II, GV = gametocytes stage V, BS= mixed blood stages, ooSpz = oocyst sporozoites, sgSpz= salivary gland sporozoites, LS24/LS40/LS50 = liver stages 24, 40 and 50h after invasion.

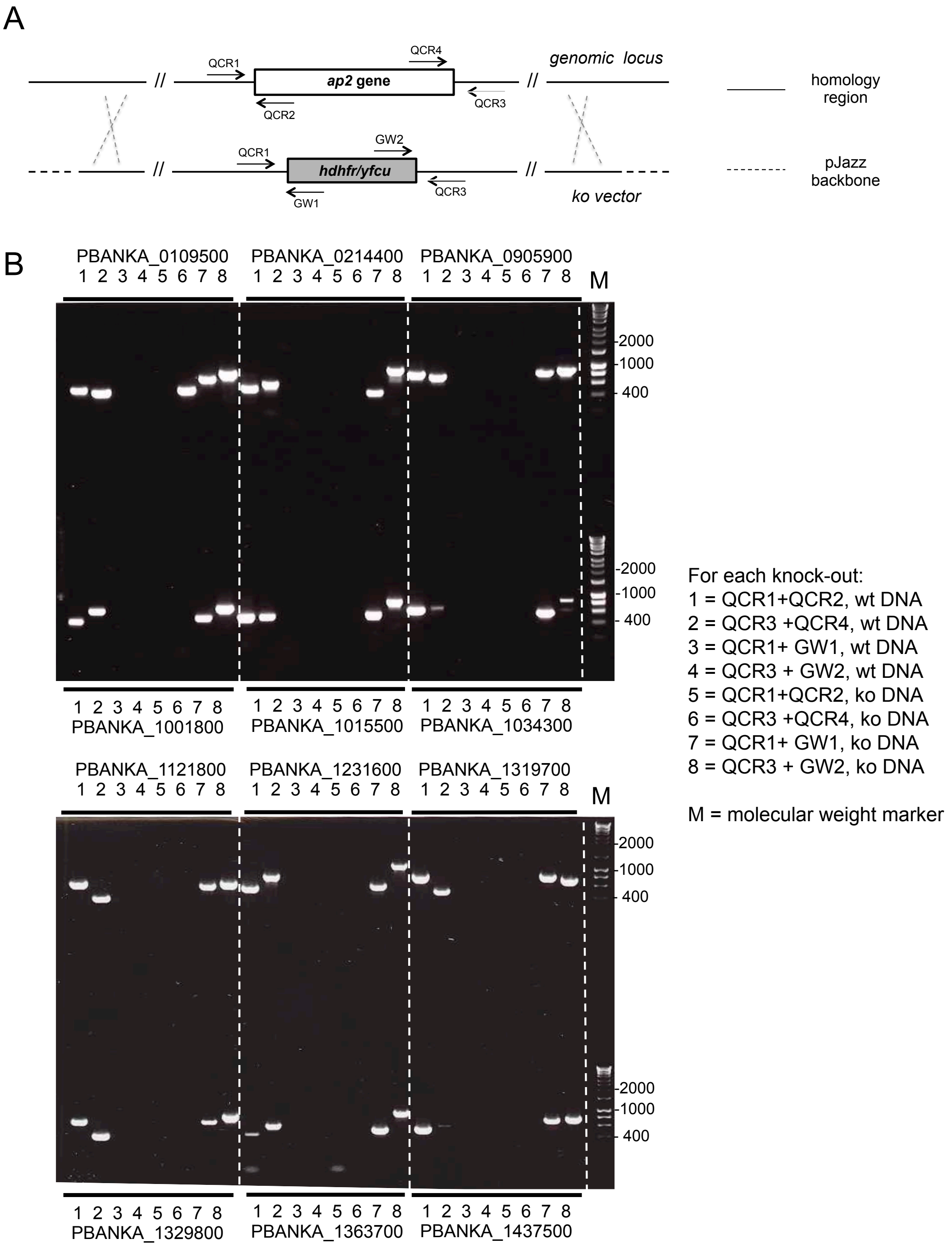

Figure S2, related to Figure 1. Generation and genotyping of ApiAP2 knockout mutants.

A) Schematic representation of a generic ApiAP2 locus, the corresponding transfection vector and annealing sites for genotyping primers. *hdhfr/yfcu* = positive/negative selection cassette.

B) Agarose gel images showing PCR products from genotyping a representative knockout clone for each gene disrupted in this study. The genotyping panel for each gene consists of four PCR reactions performed on wt and mutant gDNA as described in the side panel. Transfections with a knockout vector for PBANKA_0109500 also repeatedly generated PCR-positive parasites, but cloning was unsuccessful and a PCR product indicative of an undisrupted gene locus was retained (lane 6).

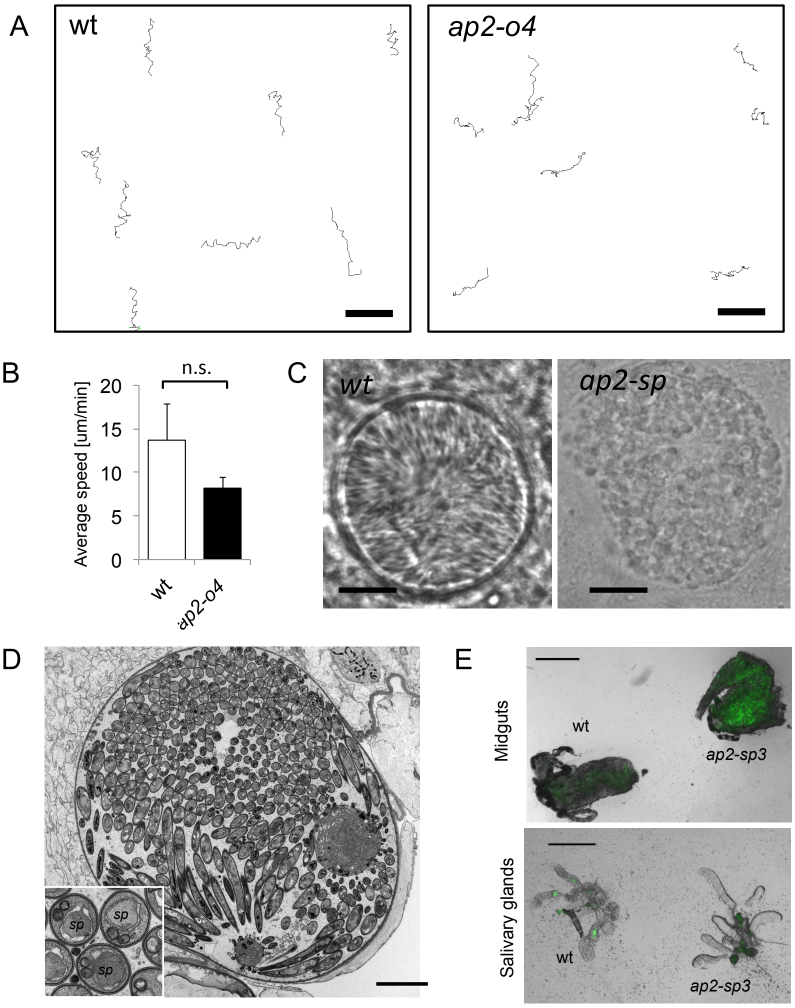

Figure S3, related to Figures 2 and 3. Additional phenotyping of *ap2-o4, ap2-sp* and *ap2-sp3* parasites.

A) Motility traces generated by manual tracking of ookinetes from time lapse movies acquired for 10 min at 1 frame every 10s. Typical helical gliding patterns of ookinetes in matrigel are shared by wild type and *ap2-o4* ookinetes. Scale bar = 75 µm.

B) Gliding speed of wt and *ap2-o4* ookinetes in Matrigel determined by time-lapse video microscopy. Error bars show standard deviations for 25 wild type and 19 mutant ookinetes.

C) Comparison of the development of the *ap2-sp* and wt oocysts on day 14 post infections. While the developing sporozoites can be observed in the wt oocysts, the *ap2-sp* ones are undergoing the abnormal granulation as described previously (Yuda et al., 2010). Scale bar = 10 µm.

D) Transmission electron micrograph of a representative *ap2-sp3* oocyst on a mosquito midgut on day 12 post feeding, showing normal sporozoite budding (compare to wild type oocysts in Fig. 3D). Scale bar = 5 µm.

E) Representative light microscopic images of midguts and salivary glands overlaid with fluorescence images to reveal parasites, which express GFP. Organs were dissected from the same two mosquitoes 21 days after infection with wild type or *ap2-sp3* parasites. Wild type parasites have progressed from the midgut to the salivary glands, while mutant parasites appear to be retained within intact oocysts on the midgut and fail to migrate to the salivary glands. Scale bar = 75 µm.

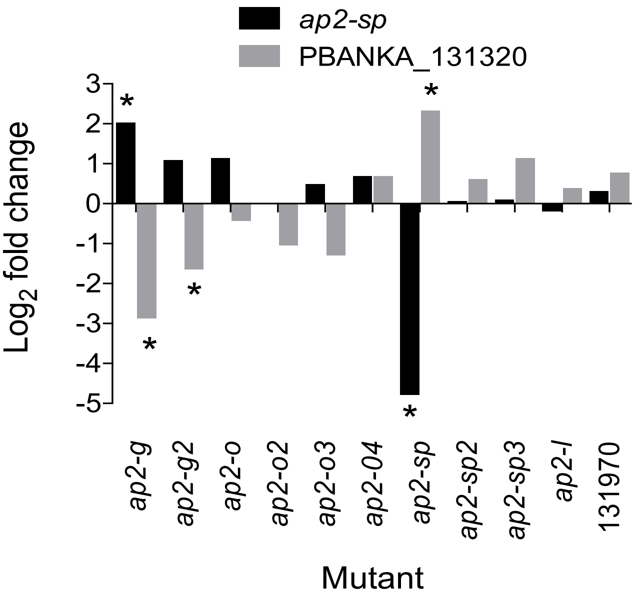

Figure S4 related to Figure 4. PBANKA_1313200 as potential secondary regulator connected with *ap2-sp* expression

Differential expression of *sp2-sp* and PBANKA_1313200 in synchronous blood stage cultures of mutant clones expressed relative to wild type. Asterisks indicate all instances of statistical significance (adjusted p-values < 0.05, n = 3 biological replicates).

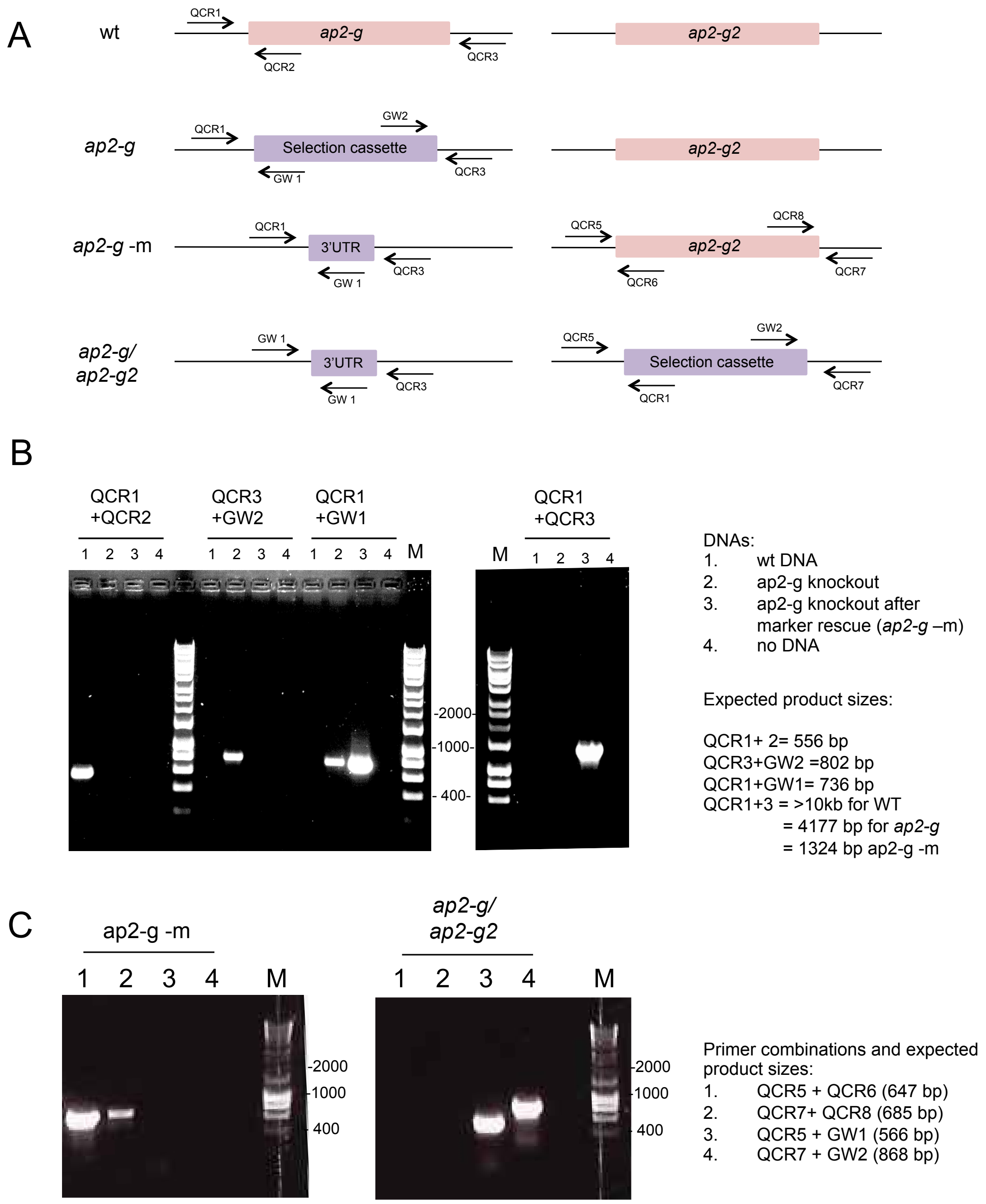

Figure S5, related to Figure 6. Generation and genotyping of a *ap2-g/ap2-g2* double mutant.

A) Schematic representation of *ap2-g* and *ap2-g2* loci in wild type and mutant parasite including marker free intermediate (*ap2-g* -m), showing annealing sites of genotyping primers. QCR1-4 sequences are the same as for genotyping the *ap2-g* single knockout. QCR5-8 sequences correspond to QCR1-4 of *ap2-g2* genotyping set.

B) Agarose gel images showing PCR products that confirm loss of the selection marker from an *ap2-g* –m clone following negative selection.

C) PCR products confirming modification of the *ap2-g2* locus in an *ap2-g/ap2-g2* double knockout.

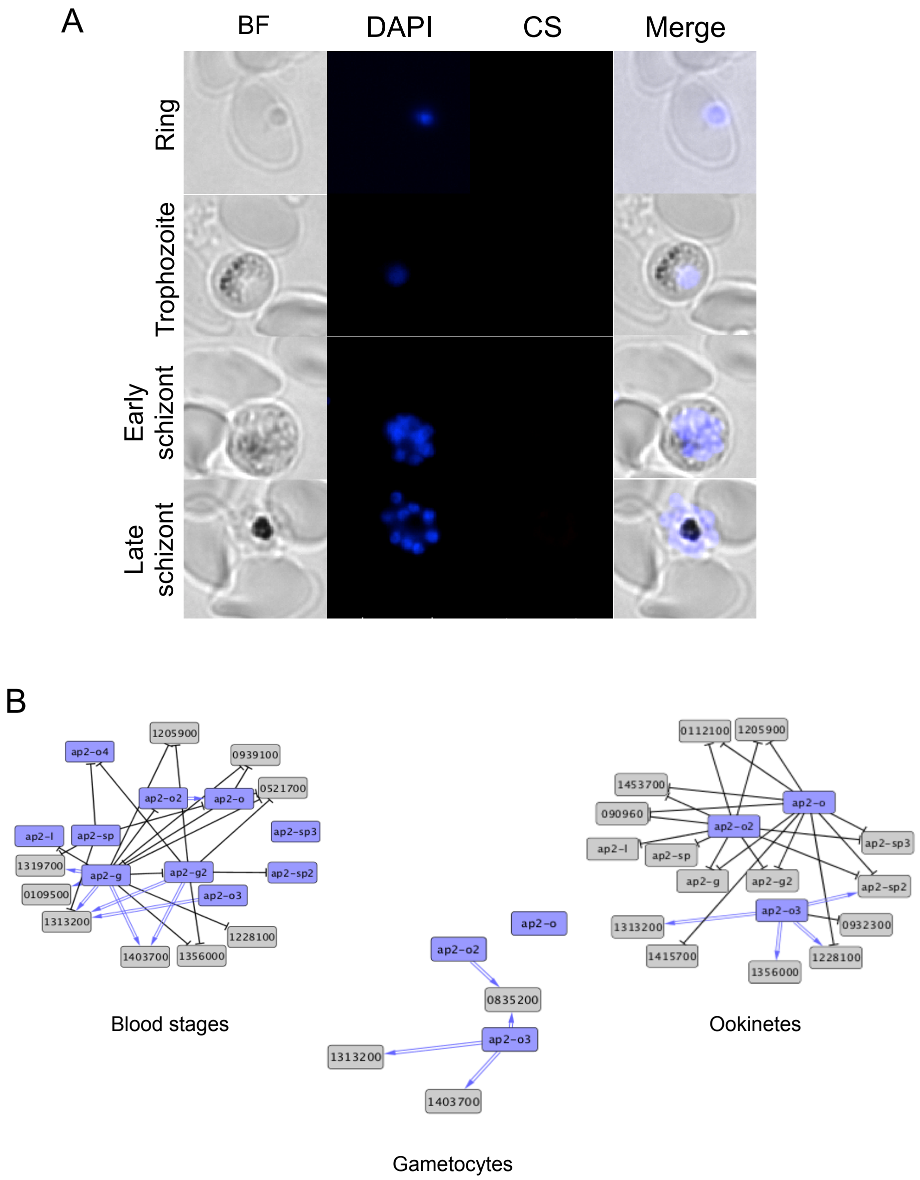

Figure S6, related to Figures 6 and 4. Lack of CSP expression in the *ap2-g* parasite line before the introduction of *ap2-g2 ko* mutation and network representation of ApiAP2 gene expression.

A) The *ap2-g* parasites processed and imagined in parallel with *the ap2-g/ap2-g2* parasites presented in Fig 6 C.

B) Changes in the expression of ApiAP2 genes in the generated mutants mutants. The Blue nodes show disrupted genes, edges show the resulting expression changes with $p<0.05$. Blue double arrows indicate positive interactions (i.e. the target gene is less expressed in the mutant) and black lines show negative interactions. The diagrams illustrate a strong interdependence of ApiAP2 genes, but interactions shown are not necessarily direct.

Supplemental data items:

Data S1, related to Figure 1. Summary of the apiAP2 knockout screen and phenotyping of the generated mutants.

Data S2, related to Figure 4. Transcriptome analysis of the apiAP2 mutants.

Data S3, related to Figure 4 and 5. Groups of genes co-expressed across different apiAP2 mutants.

Data S4, related to Figure 4 and 5. Characterization of the 49 co-expression clusters generated from ApiAP2 expression data.

Data S5, related to Figure 6. Transcriptome and proteome analysis of the double *ap2-g/ap2-g2* knockout.

Supplemental experimental protocols:

Growth rate quantification using barseq approach. The relative fitness associated with *Plasmo*GEM knockout vectors was quantified as described previously (Gomes et al., 2015). Briefly, a mixed pool containing 14 barcoded ApiAP2 ko vectors and 7 control vectors was prepared and used for three independent transfections of a schizont culture prepared from a Wistar rat (~1% parasitaemia). Electroporated parasites were injected intravenously into BALB/c mice, and resistant parasites were selected with 70 mg/ml of pyrimethamine in the drinking water. Infections were monitored daily using Giemsa stained thin blood smears. Blood samples were taken on days 4-8 and used for genomic DNA extractions with phenol/chloroform. Vector specific barcodes of 10-11 bp were PCR-amplified using a generic primer pair and the amplicons were converted into sequencing libraries by PCR-mediated Illumina adapter addition as described (see Gomes et al., 2015). Samples were pooled and sequenced on an Illumina MiSeq sequencer (150 bp paired-end reads). A perl script was used to extract and count the barcodes from the sequencing output. The barcodes corresponding to vectors not included in the input or accounting for less than 0.1% of barcode reads obtained from a given sample were excluded from further analysis. The number of reads corresponding to each barcode was corrected for library size and the relative fitness of each vector on each day was calculated by dividing the fold change of the relative abundance of the vector tag by the averaged results from four reference genes with normal growth rates.

PCR genotyping of the generated mutants. Each mutant clone was genotyped using four combinations of primers, specific for either wt or ko locus on both sides of the targetted gene (schematic design shown in Fig. S2). Wt DNA control was included in each genotyping panel. The details of DNA preparation and PCR program can be found on http://plasmogem.sanger.ac.uk/info/protocols.

Following primers were used:

QCR1	
PBANKA_0109500	ACTTAATATTTATACACTGCGTTTTCTC
PBANKA_0214400	TATAGTGCGGATGTATAGCACACG
PBANKA_0905900	TTACATATATGGAAAATAATGAGCACC
PBANKA_1001800	TATCATGTCAACATATCATCATATTGC
PBANKA_1015500	CACCAAATTGTGCATCTTTCG
PBANKA_1034300	ATTAATAAACTAGAGGTTCTTTACCAGG
PBANKA_1121800	TGTGGATGAAAATGTGGTTGC
PBANKA_1231600	ATTATCATTGTGGATTAACGGAAG
PBANKA_1319700	ATAGACTGTGTCAATAGATGATTTCAGC
PBANKA_1329800	TTATGCCTGTATTTGAAACGAGG
PBANKA_1363700	CGAACAAATATATAAATGCGAAGAGG
PBANKA_1437500	AGGGGCATTGAACAATAGCACCT

QCR2	
PBANKA_0109500	TAAGAAAAAGTTAGACCAAATAATGAGG
PBANKA_0214400	AAATAATATACACAATATGAGAATCCAAG
PBANKA_0905900	AGAGGCATCTATATTCAAATTGGC
PBANKA_1001800	TTTTCAAATAATGGGAATACAGTGG
PBANKA_1015500	AAAGATTCTATACATTCAGACACTTTACAC
PBANKA_1034300	TAAATCAGTGAAATCAACTTATGGATC
PBANKA_1121800	CAAAGCAATAATAGTTATATAACGGAAGG
PBANKA_1231600	CGAGACAATTGTTTATCAAATTTAGC
PBANKA_1319700	AGGATATTATGATCTTGAGATTGATGG
PBANKA_1329800	GGAAGTAGAACAGGAATAGTAACTGACC
PBANKA_1363700	TATTTCGTTCAAGGTTTTCCCTC
PBANKA_1437500	TGGCGTTGAAACTAGTCCCGA
QCR3	
PBANKA_0109500	ATAATCGTAAAACTCGTTCAAAGG
PBANKA_0214400	AGAAGCATAGTTGTAAATTCATCAAATACTG
PBANKA_0905900	AACAAATTAAATGCATTGATTAGACAG
PBANKA_1001800	CTGTACATATTTTGAAGCGATGATGTAC
PBANKA_1015500	TCAACGACAGACATTGAAAAGC
PBANKA_1034300	GTTATACTATTTCGATGTAA
PBANKA_1121800	CATTTGTTTTTATGTACATGAGTATAAGTGC
PBANKA_1231600	CCATGTGTATGTTATTATATAAGAAGAATG
PBANKA_1319700	TGATAATTATTATACAGTTGTGCCGC
PBANKA_1329800	CACATATAGTCATATATATATACCGATAAGAAGG
PBANKA_1363700	TTGATTAAAGAAATCTCACAACATAAACAG
PBANKA_1437500	CATTTCAATTGCAACGGG
QCR4	
PBANKA_0109500	AATTTGTGTGCAAATAGCTATACATG
PBANKA_0214400	TCACATCGGTTATCTATTTCATTTC
PBANKA_0905900	TTAGAATTACCAGATAAAGATGGACC
PBANKA_1001800	TTTACAACGTACCACTCCTCCTG
PBANKA_1015500	TCGGACCACCTTGCTTCC
PBANKA_1034300	CTTTCAGAATGAAGGAAG
PBANKA_1121800	CCAAATACTTTTCCTTTTATCTTGG
PBANKA_1231600	GCTATATCATATGAATATGTATTAATAGGTGC
PBANKA_1319700	TTACATCTTATATCATCCTTATTCTGGAG
PBANKA_1329800	CTGTTAATAAGTTCGGCATTTATCG
PBANKA_1363700	TCTATCAAGGATCTTATGTAGCTAACTGG
PBANKA_1437500	AGAATATTTCATACCATATG
Cassette specific primers	
GW1	ACTTAATATTTATACACTGCGTTTTCTC
GW2	TATAGTGCGGATGTATAGCACACG

Ookinete motility assays. For the motility assay 30 µl of 22 h ookinete culture were mixed with 30 µl Matrigel (BD Biosciences) on ice. A drop of the mixture was spotted on a microscope slide, covered with a coverslip, sealed with nail polish and allowed to settle for ~30 min at 19°C. A Leica M205A microscope was used to acquire time-lapse movies (1 frame every 10 s for 10 min) of representative fields containing at least five ookinetes. The results were exported as *.tiff image series and analysed using Fiji software with the Manual Tracking plugin (http://pacific.mpi-cbg.de/wiki/index.php/Manual_Tracking).

Immunofluorescence imaging. Infected red blood cells were washed with phosphate buffered saline (PBS), fixed with 4% paraformaldehyde in PBS (20 min), permeabilized with 0.01% Triton X-100/PBS solution (10 min) and blocked with 3% bovine serum albumin in PBS for 1 h. Cy3 conjugated anti-PbCSP D11 monoclonal antibody (Yoshida et al., 1980) diluted with blocking solution to 1 µg/ml was incubated with the sample for 1 h in the dark. The cells were washed 3 times with 1xPBS and mounted on a microscope slide using ProLong Gold Antifade Mountant with DAPI stain (Life Technologies). Samples were imaged using a fluorescence microscope (Leica DMi8) with a digital camera (Leica DFC265Fx).

Transmission electron microscopy. For each strain the midguts from 10 mosquitoes were dissected and the presence of oocysts verified using fluorescence microscopy. Five highly infected guts were fixed at 20 °C for 2 h in 2% PFA with 2.5% glutaraldehyde in 0.1 M sodium cacodylate buffer at pH 7.42 and then in 1% buffered osmium tetroxide for another 2 h, mordanted with 1% tannic acid for 1 h and dehydrated through an ethanol series staining en bloc with 2% uranyl acetate at the 30% stage. The midguts were oriented and embedded in Epon resin for ultrathin sectioning on a Leica UC6 microtome, stained with uranyl acetate and lead citrate and analysed on a 120 kV Spirit Biotwin transmission electron microscope. Images were taken on a Tietz F4.15 CCD camera.

Parasite purification for transcriptome sequencing. To synchronise parasites at the schizont stage, phenylhydrazine-treated TO mice were injected intraperitoneally with $\sim 1\times10^7$ infected red blood cells. On day 3 post infection the presence of parasitaemia (5-15%) was confirmed using Giemsa stained thin blood smears and blood was harvested by cardiac puncture. White blood cells were removed using Plasmodipur filters (Europroxima). The parasites were cultured for 22 h and red blood cells harbouring schizonts or gametocytes were purified on a cushion of 55% Nycodenz in culture medium, as described previously (Janse et al., 2006). The resulting pellet was washed in ice cold erythrocyte lysis buffer (0.15 M NH_4Cl, 0.01 M $KHCO_3$, 0.001 M EDTA) until the supernatant was clear to remove host cell RNA, and parasites were resuspended and lysed in TRIzol reagent (Invitrogen).

To obtain highly enriched gametocytes, phenylhydrazine-treated mice were infected with $\sim 1\times10^7$ parasites, and treated with sulfadiazine by intraperitoneal injection (100 µl of 40 µg/ml solution in PBS) from day 2 post infection as well as by addition of drug to the drinking water (20 mg/l). On day 4, after verifying the absence of asexual stages, the blood was harvested by cardiac puncture and

transferred into suspended animation (SA) medium (RPMI1640 with L-glutamine, 25 mM HEPES, 4 mM $NaHCO_3$, 5% FCS, pH = 7.2), which prevents gametocyte activation. White blood cells were removed using Plasmodipur filters and the blood was layered on a cushion of 48% Nycodenz/SA buffer and spun for 15 min at 500 g (no breaks) to separate the gametocytes from the other blood cells. The gametocyte layer was harvested, pelleted and resuspended in TRIzol reagent.

For ookinete samples the animals were infected and drug treated as for gametocyte preparations. To initiated gamete formation and fertilization, gametocyte infected blood from 1 mouse was added to 30 ml of the ookinete medium (RPMI1640 containing 25 mM HEPES, 10% FCS, 100 µM xanthurenic acid, pH 7.5) and cultured at 19 °C. After 22 h parasites were purified using MACS CS magnetic separation columns (Miltenyi Biotec) according to manufactures instructions, with ookinete medium used for column conditioning, washes and elution. The ookinetes were spun down at 500 g and resuspended in TRIzol reagent. TRIzol samples from all parasite stages were left at room temperature for 10 min and then either frozen at -80 °C or used directly for RNA extraction.

RNA extraction and RNA-seq library preparation. Total RNA was extracted using standard chloroform extraction and isopropanol precipitation (Chomczynski, 1993). RNA concentration and integrity was measured on an Agilent Bioanalyzer using the RNA 6000 Nano kit and on a NanoDrop 1000 spectrophotometer. 1-2 µg of total RNA from each sample was used for mRNA isolation (Magnetic mRNA Isolation Kit, NEB). First strand cDNA synthesis was performed using the SuperScript III First-Strand Synthesis System and a 1:1 mix of Oligo(dT) and random primers (Invitrogen). The DNA/RNA hybrids were purified using Agencourt RNAClean XP beads (Beckman Coulter) and the second cDNA strand was synthetized using a 10 mM dUTP nucleotide mix, DNA Polymerase I (Invitrogen) and RNAseH (NEB) for 2.5 h at 16 °C. The long cDNA fragments were purified and fragmented using a Covaris S220 system (duty cycles = 20, intensity = 5, cycles/burst = 200, time = 30 s). The ~200 bp long fragments were end-repaired, dA-tailed and ligated to "PCR-free" adapters (Kozarewa et al., 2009) with index tags using NEBNext kits according to the manufacturer's instructions. Excess adapters were removed by two rounds of clean-up with 1x volume of Agencourt AMPure XP beads. Final libraries were eluted in 30 µl water, quality-controlled using Agilent High Sensitivity DNA chip, digested with USER enzyme (NEB) and quantified by qPCR. For some libraries additional 5 cycles of PCR amplification were performed, using KAPA HiFi HotStart PCR mix and Illumina tag-specific primers to obtain enough material for sequencing. Pools of ~12 indexed libraries were sequenced using an Illumina HiSeq2500 system (100 bp paired-end reads).

RNAseq data processing. Sequence reads underwent basic quality control (quality score, GC content, adapter contamination), adapter clipping and library segregation according to the multiplexed tags, using standard data processing pipelines at the Wellcome Trust Sanger Institute. The filtered reads were downloaded as *.cram files aligned to the *P. berghei* ANKA reference genome and transformed using Scramble (Bonfield, 2014) and Picard tools (http://broadinstitute.github.io/picard/). The resulting unaligned *.fastq files were mapped against the *P. berghei* transcriptome (PlasmoDB version 9.3)

using Tophat2 aligner (Kim et al., 2013) set to “fr-firststrand” for library type and a maximum intron size of 15 kb. The re-aligned *.bam files were sorted and indexed using SAMtools suite (Li et al., 2009) and used both for visual data inspection in the Artemis genome browser (Rutherford et al., 2000), and to generate read count tables for all annotated *P. berghei* transcripts with HTSeq Python package (Anders et al., 2014).

R 3.0 software with the DEseq2 package (Love et al., 2014) was used for principal component and differential expression analyses. To cluster analysis, reads were first normalized for library size and genes with <50 reads across all samples were removed. For each remaining gene the average number of reads at each strain/stage combination was divided the sum of averaged reads from all conditions, generating the proportional expression profile of the gene. The expression profiles were clustered using Ward’s hierarchical clustering with a cutoff set to 49 clusters.

The Gene Ontology (GO) terms enrichment was performed using PlasmoDB (http://plasmodb.org). For metabolic pathways the data from the manually curated Malaria Parasite Metabolic Pathways database (mpmp.huji.ac.il); (Ginsburg, 2006) was translated to *P. berghei* orthologs, and Fisher’s exact test with multiple testing correction was used to test for the enrichment. Additionally custom datasets were downloaded from the literature and tested in the same way. These included: genes encoding male- and female- specific proteins (Tao et al., 2014), transcripts overrepresented at more than one time points during liver development compared with asexual blood stages (translated from *P. yoelii* orthologs) (Tarun et al., 2008), targets for AP2-O binding in ookinetes (Kaneko et al., 2015) and mRNAs bound by DOZI/CITH translational repression complex in female gametocytes (Guerreiro et al., 2014).

LC-MS/MS analysis. Three independent schizonts cultures from *ap2-g/ap2-g2* and *ap2-g* parasites were prepared and parasites were isolated as described in the transcriptome analysis section. Each parasite pellet was lysed in 300 µL RIPA buffer with 1x phosphatase and proteinase inhibitor (Thermo Fisher) on ice, then TCEP was added to final ~40 mM and heated at 56 °C for 15 min. The suspension was processed in ultrasonic bath then centrifuged at 14,000 rpm to collect the supernatant. To the pellet formed, 200 µL of 8 M urea/40 mM TCEP/50 mM TEAB was added sonicated and centrifuged as above. Both supernatants were pooled, and the protein concentrations were measured by 660 nm Protein Assay (Thermo Fisher). Iodoacetamide was then added to the sample for alkylation. 90 µg of protein was taken from each sample and made up to the same volume and concentration with 100 mM TEAB (Fluka). Chloroform/methanol precipitation was performed to remove detergent. The protein pellet was resuspended in 90 µL of 100 mM TEAB and digested with 3 µg trypsin (Gold, Promega) at 37°C for 12 h, then labelled with TMT10plex (Thermo Fisher) as described in the product instruction. Ten labelled samples were then pooled to one and dried in a SpeedVac.

The peptides were fractionated on an XBridge BEH C18 column (4.6 mm i.d. x 250 mm, 130 Å, 3.5 µm, Waters) at a high pH reverse phase (pH ~ 10.5 by NH_4OH solution) at a 30 s intervals. Fractions were then pooled into 16 pools and dried in a SpeedVac. Peptides were resuspended in 0.5% formic

acid (FA)/100% H_2O, and ¼ was injected for the LC-MS/MS analysis on an Orbitrap Fusion coupled with an Ultimate 3000 RSLCnano instrument. The peptides were first loaded to a peptide trap (100 µm i. d. x 20 mm, 100 Å, 5 µm) then separated on a nano-analytical column (75 µm i. d. x 500 mm) at a linear gradient of 4-32% ACN/0.1% FA within 120 min. The Orbitrap Fusion was operated at a TopSpeed method with a 3 s cycle time, while the full MS scans were acquired at 120,000 resolution at m/z 200; the MS2 spectra were acquired in ion trap mode with CID fragmentation with the isolation window set to m/z 0.7; and finally the MS3 spectra were acquired in Orbitrap at a resolution of 60,000 (m/z 200) using a multinotch MS3 strategy and HCD fragmentation with the isolation window at m/z 0.5. The data have been deposited to the ProteomeXchange Consortium via the PRIDE partner repository with the dataset identifier PXD005180.

All LC-MS data were processed in Proteome Discoverer 1.4 using the SequestHT to search a combined protein database of *P. berghei* (http://plasmodb.org/) and mouse (UniprotKB). Peptide spectral matches (PSM) were filtered to a false discovery rate (FDR) of 1%, and isobaric mass tags were used to calculate the sample/reference ratios for each sample. Host and *Plasmodium* proteins with PSM ratio counts < 3 were removed from further analysis. For the remaining proteins, sample/reference ratios from all unique peptides were averaged and normalised within each sample. A two-tailed T-test on log2 transformed ratios was used to identify the significantly up- and down regulated proteins.

Supplemental references:

Anders, S., Pyl, P.T., and Huber, W. (2014). HTSeq - A Python framework to work with high-throughput sequencing data. Bioinformatics *31*, 166–169.

Bonfield, J.K. (2014). The Scramble conversion tool. Bioinformatics *30*, 2818–2819.

Chomczynski, P. (1993). A reagent for the single-step simultaneous isolation of RNA, DNA and proteins from cell and tissue samples. Biotechniques *15*, 532–534, 536–537.

Ginsburg, H. (2006). Progress in in silico functional genomics: the malaria Metabolic Pathways database. Trends Parasitol. *22*, 238–240.

Gomes, A.R., Bushell, E., Schwach, F., Girling, G., Anar, B., Quail, M.A., Herd, C., Pfander, C., Modrzynska, K., Rayner, J.C., et al. (2015). A genome-scale vector resource enables high-throughput reverse genetic screening in a malaria parasite. Cell Host Microbe *17*, 404–413.

Guerreiro, A., Deligianni, E., Santos, J.M., Silva, P.A., Louis, C., Pain, A., Janse, C.J., Franke-Fayard, B., Carret, C.K., Siden-Kiamos, I., et al. (2014). Genome-wide RIP-Chip analysis of translational repressor-bound mRNAs in the Plasmodium gametocyte. Genome Biol. *15*, 493.

Janse, C.J., Ramesar, J., and Waters, A.P. (2006). High-efficiency transfection and drug selection of genetically transformed blood stages of the rodent malaria parasite Plasmodium berghei. Nat. Protoc. *1*, 346–356.

Kaneko, I., Iwanaga, S., Kato, T., Kobayashi, I., and Yuda, M. (2015). Genome-Wide Identification of the Target Genes of AP2-O, a Plasmodium AP2-Family Transcription Factor. PLoS Pathog. *11*, e1004905.

Kim, D., Pertea, G., Trapnell, C., Pimentel, H., Kelley, R., and Salzberg, S.L. (2013). TopHat2: accurate alignment of transcriptomes in the presence of insertions, deletions and gene fusions. Genome Biol. *14*, R36.

Kozarewa, I., Ning, Z., Quail, M.A., Sanders, M.J., Berriman, M., and Turner, D.J. (2009). Amplification-free Illumina sequencing-library preparation facilitates improved mapping and assembly of (G+C)-biased genomes. Nat. Methods *6*, 291–295.

Li, H., Handsaker, B., Wysoker, A., Fennell, T., Ruan, J., Homer, N., Marth, G., Abecasis, G., and Durbin, R. (2009). The Sequence Alignment/Map format and SAMtools. Bioinformatics *25*, 2078–2079.

López-Barragán, M.J., Lemieux, J., Quiñones, M., Williamson, K.C., Molina-Cruz, A., Cui, K., Barillas-Mury, C., Zhao, K., and Su, X. (2011). Directional gene expression and antisense transcripts in sexual and asexual stages of Plasmodium falciparum. BMC Genomics *12*, 587.

Love, M.I., Huber, W., and Anders, S. (2014). Moderated estimation of fold change and dispersion for RNA-seq data with DESeq2. Genome Biol. *15*, 550.

Otto, T.D., Böhme, U., Jackson, A.P., Hunt, M., Franke-Fayard, B., Hoeijmakers, W.A.M., Religa, A.A., Robertson, L., Sanders, M., Ogun, S.A., et al. (2014). A comprehensive evaluation of rodent malaria parasite genomes and gene expression. BMC Biol. *12*, 86.

Rutherford, K., Parkhill, J., Crook, J., Horsnell, T., Rice, P., Rajandream, M.A., and Barrell, B. (2000). Artemis: sequence visualization and annotation. Bioinformatics *16*, 944–945.

Tao, D., Ubaida-Mohien, C., Mathias, D.K., King, J.G., Pastrana-Mena, R., Tripathi, A., Goldowitz, I., Graham, D.R., Moss, E., Marti, M., et al. (2014). Sex-partitioning of the Plasmodium falciparum stage V gametocyte proteome provides insight into falciparum-specific cell biology. Mol. Cell. Proteomics *13*, 2705–2724.

Tarun, A.S., Peng, X., Dumpit, R.F., Ogata, Y., Silva-Rivera, H., Camargo, N., Daly, T.M., Bergman, L.W., and Kappe, S.H.I. (2008). A combined transcriptome and proteome survey of malaria parasite liver stages. Proc. Natl. Acad. Sci. U. S. A. *105*, 305–310.

Yoshida, N., Nussenzweig, R.S., Potocnjak, P., Nussenzweig, V., and Aikawa, M. (1980). Hybridoma produces protective antibodies directed against the sporozoite stage of malaria parasite. Science *207*, 71–73.

Yuda, M., Iwanaga, S., Shigenobu, S., Kato, T., and Kaneko, I. (2010). Transcription factor AP2-Sp and its target genes in malarial sporozoites. Mol. Microbiol. *75*, 854–863.
