# Supplementary material for: A Knockout Screen of ApiAP2 Genes Reveals Networks of Interacting Transcriptional Regulators Controlling the Plasmodium Life Cycle
Source: Cell Host Microbe. 2017 Jan 11;21(1):11–22. doi: 10.1016/j.chom.2016.12.003 (PMC5241200; doi:10.1016/j.chom.2016.12.003)
Supplement: Data S4. Characterization of the 49 Co-expression Clusters Generated from ApiAP2 Expression Data — Related to Figures 4 and 5. [file mmc5.zip › Data_S4.pdf]

	Expression profile	N	Enrichment analysis (MPMP and additional datasets)(C)	Motif enriched within the cluster
Cluster 1	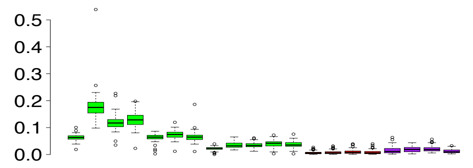	78	Characteristics of Plasmodium falciparum export proteins that remodel infected erythrocyte (P=5.64e−06)<br>Genes coding for components involved in ribosome assembly (P=3.93e−05)<br>Maturation and export of 60S and 40S ribosomal subunits (P=3.93e−05)<br>Translocon of exported proteins (PTEX) (P=5.32e−05)	none
Cluster 2	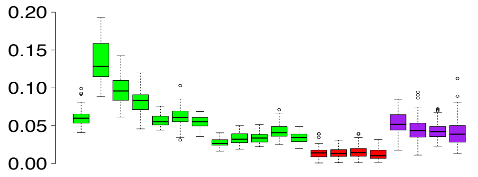	74	Genes coding for components involved in ribosome assembly (P=2.13e−16)<br>Maturation and export of 60S and 40S ribosomal subunits (P=1.57e−10)	none
Cluster 3	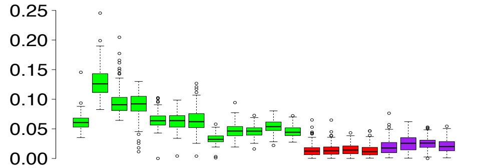	142	Genes coding for components involved in ribosome assembly (P=8.53e−04)<br>Maturation and export of 60S and 40S ribosomal subunits (P=2.71e−03)	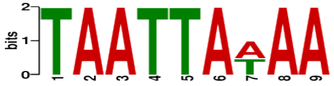 E = 3.0e-003
Cluster 4	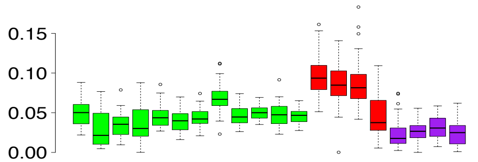	50	none	none
Cluster 5	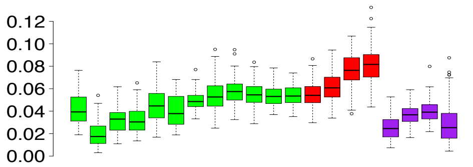	88	DNA Replication (P=1.47e−04)<br>Genes coding for enzymes/proteins involved in DNA replication (P=5.72e−06)<br>Genes involved in excision−repair (P=1.09e−03)<br>Nucleotide excision repair (P=1.48e−03)<br>Pre−replicative complex formation and transition to replication (P=3.11e−04)	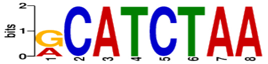 E = 3.6e-007<br>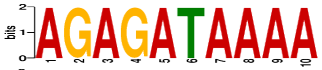 E = 3.1e-002<br>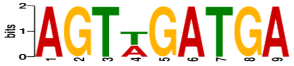 E = 3.4e-002
Cluster 6	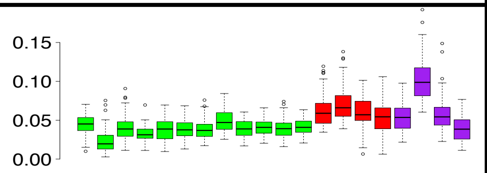	68	Protein kinase coding genes<br>(P=1.11e−03)	none

	Expression profile	N	Enrichment analysis (MPMP and additional datasets)(C)	Motif enriched within the cluster
Cluster 7	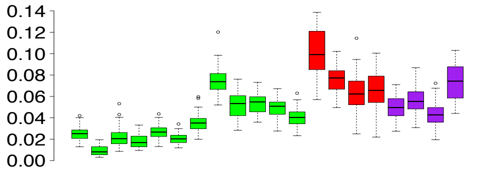	45	none	none
Cluster 8	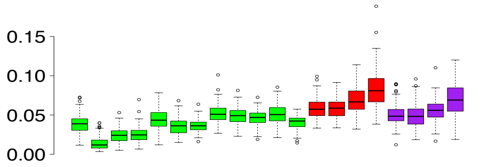	164	Male_specific genes (P=1.54e−04)<br>Centriole proteins (P=1.14e−02)<br>Kinetochores power chromosome movements in mitosis (P=1.14e−02)<br>Proteins involved in steps during passage through prophase (P=1.47e−02)<br>Structure of the mitotic centrosome (P=1.14e−02)	none
Cluster 9	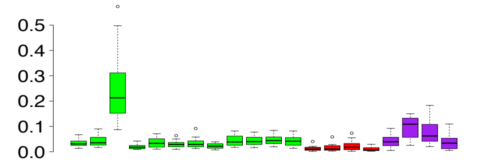	16	Liver stage enriched transcripts (P=8.77e−07)<br>Pyruvate metabolism (P=7.79e−03)	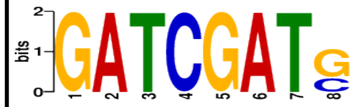 E = 2.9e-002
Cluster 10	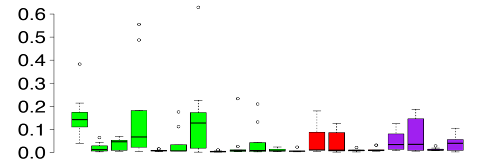	9	none	none
Cluster 11	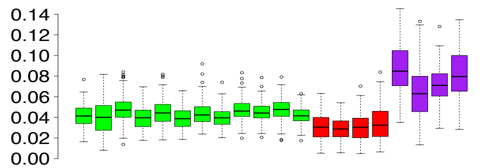	135	none	none
Cluster 12	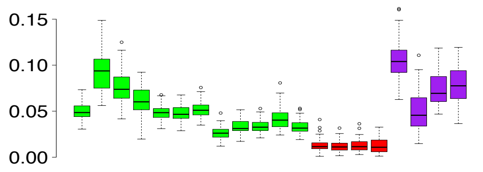	42	AP2-O targets (P=7.18e−03)<br>Genes coding for components involved in ribosome assembly (P=4.32e−06)<br>Maturation and export of 60S and 40S ribosomal subunits (P=1.05e−05)<br>Pre−ribosomal particles along the 60S assembly pathway (P=5.87e−03)	none

	Expression profile	N	Enrichment analysis (MPMP and additional datasets)(C)	Motif enriched within the cluster
Cluster 13	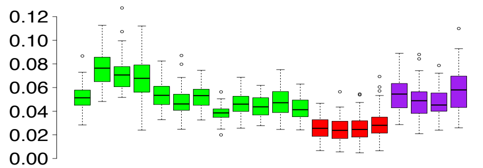	71	none	none
Cluster 14	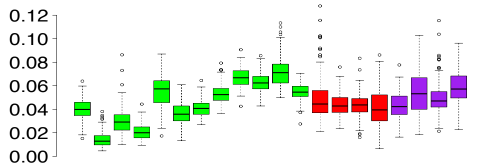	132	none	none
Cluster 15	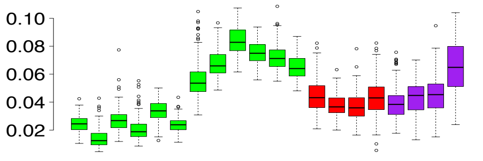	124	Mitochondrial electron flow (P=3.5e−02)<br>Proteasome−mediated proteolysis of ubiquinated proteins (P=3.5e−02)<br>S−Glutathionylated proteins (P=3.5e−02)<br>The acidocalcisome (P=3.5e−02)<br>Female_specific genes (P=4.5e−02)	none
Cluster 16	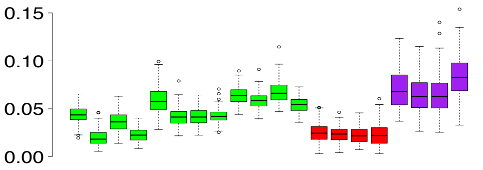	161	none	none
Cluster 17	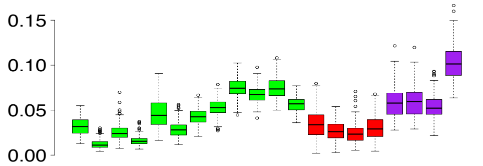	208	Female specific genes (P=4.13e−03)<br>ATP synthase complex (P=2.04e−02)	none
Cluster 18	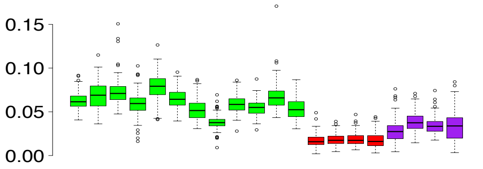	136	Parasite encoded proteins associated with the membrane of infected erythrocytes (P=8.58e−03)<br>Ribosomal structure (9.15-19)<br>Proteins of detergent-resistant membranes (2.86e-02)	none

	Expression profile	N	Enrichment analysis (MPMP and additional datasets)(C)	Motif enriched within the cluster
Cluster 19	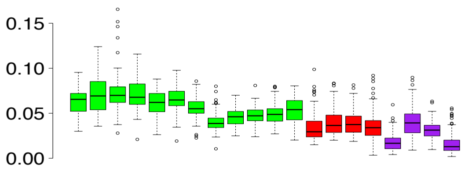	118	none	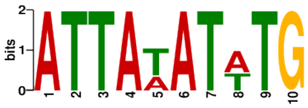 E = 2.3e-002
Cluster 20	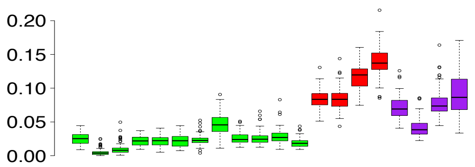	249	none	none
Cluster 21	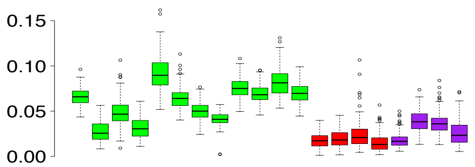	169	Nuclear genes with apicoplast signal sequences (P=2.32e−02)	none
Cluster 22	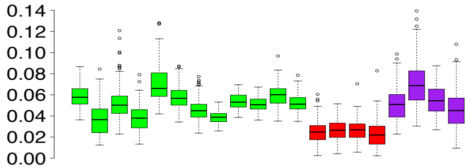	148	Initiation of translation (P=2.4e−02)	none
Cluster 23	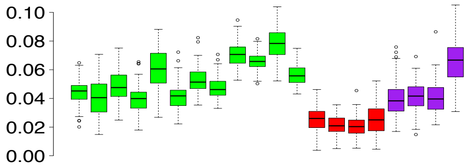	99	Ribosomal structure (P=3.22e−13)	none
Cluster 24	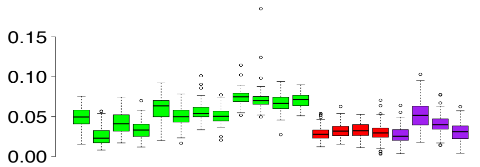	137	none	none

	Expression profile	N	Enrichment analysis (MPMP and additional datasets)(C)	Motif enriched within the cluster
Cluster 25	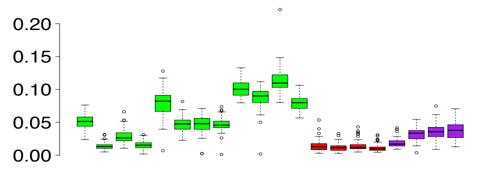	65	none	none
Cluster 26	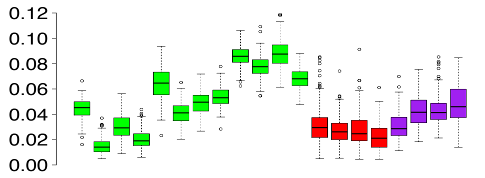	172	none	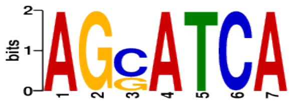 E = 2.4e-002
Cluster 27	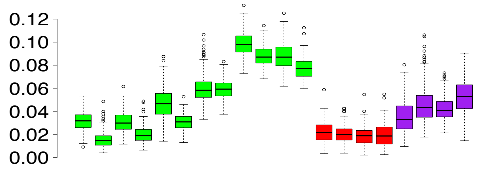	213	Classical clathrin−mediated vesicular transport (P=2.03e−03)<br>Genes coding for components of the proteasome degradation machinery & their timed transcription (P=1.17 -02)<br>Genes coding for protein traffic related proteins (P=5.86e−04)<br>Glycosylphosphatidylinositol (GPI) anchor biosynthesis (P=2.25e−02)	none
Cluster 28	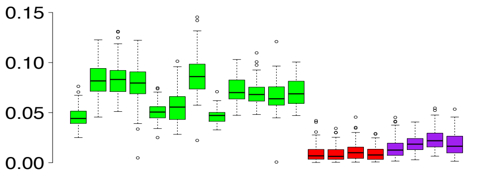	81	Functional annotation of merozoite invasion−related proteins (P=8.94e−08)<br>Genes coding for GPI−anchored membrane proteins (P=7.37e−04)<br>Subcellular localization of proteins involved in invasion (P=7.46e−09)	none
Cluster 29	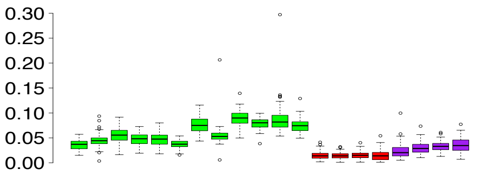	80	Protein kinase coding genes (P=1.11e−03)	none
Cluster 30	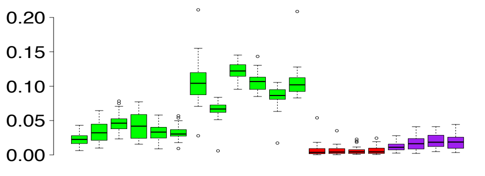	44	Established and putative Maurer.s clefts proteins (P=6.12e−04)<br>Functional annotation of merozoite invasion−related proteins (P=5.31e−12)<br>Roles of rhoptry neck proteins during invasion (P=4.58e−02)<br>Subcellular localization of proteins involved in invasion (P=5.33e−11)	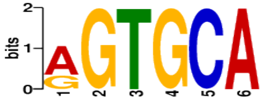 E = 1.2e-002

	Expression profile	N	Enrichment analysis (MPMP and additional datasets)(C)	Motif enriched within the cluster
Cluster 31	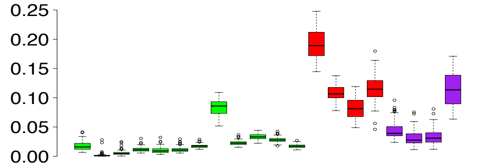	62	AP2-O targets (P=2.05e−04)<br>Translationally repressed genes (P=4.55e−44)	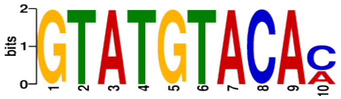 E = 2.6e-007
Cluster 32	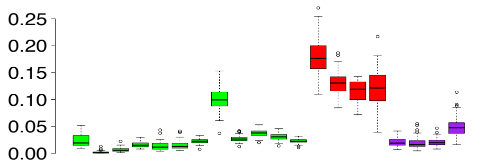	51	Translationally repressed genes (P=1.11e−22)	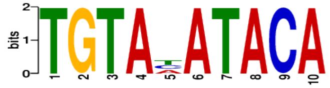 E = 1.5e-003
Cluster 33	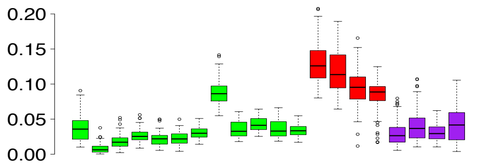	96	Translationally repressed genes (P=1.96e−10)<br>Components of the linear motor responsible for merozoite motility in invasion (P=2.73e−04)	none
Cluster 34	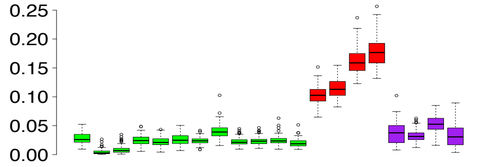	104	Male specific genes (P=2.63e−39)<br>, Molecular motor prototypes(P=1.13E-07)<br>Kinetochores power chromosome movements in mitosis (P=6.14E-06)<br>Regulation of spindle microtubule dynamics (P=1.31e-5)<br>Structure of the mitotic centrosome (P=3.73e-03)<br>Putative organization of the kinetochore (6.66e-03) ,<br>Centrosome proteins (3.76e-02)	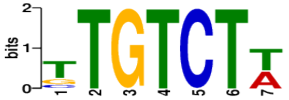 E = 2.3e-002
Cluster 35	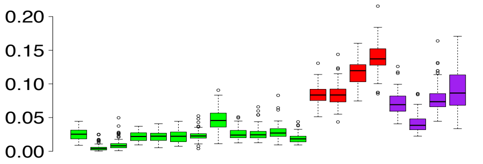	98	Male specific genes (P=7.26e−19)	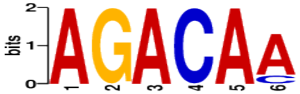 E = 7.7e-008
Cluster 36	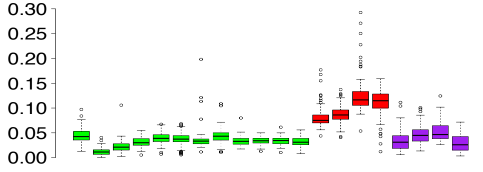	87	Male specific genes (P=4.93e−13)	none

	Expression profile	N	Enrichment analysis (MPMP and additional datasets)(C)	Motif enriched within the cluster
Cluster 37	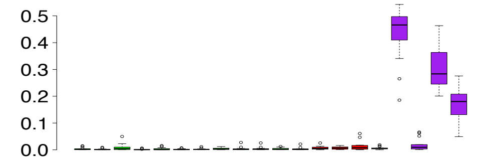	14	AP2-O targets (P=7.8e−07)	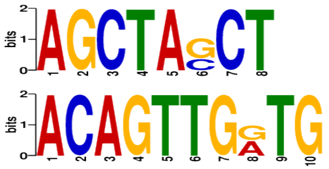 E = 9.4e-005<br>E = 1.1e-002
Cluster 38	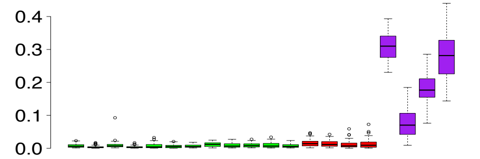	61	Role of perforin 1 in merozoite egress (P=2.37e−02)<br>AP2-O targets (P=1.55e−29)	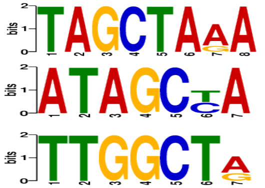 E = 8.9e-006<br>E = 5.6e-003<br>E = 1.5e-002
Cluster 39	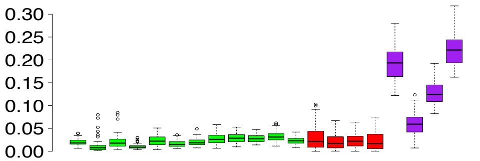	66	AP2-O targets (P=5.08e−26)	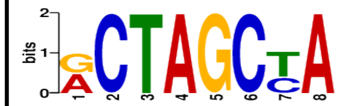 E = 1.9e-004
Cluster 40	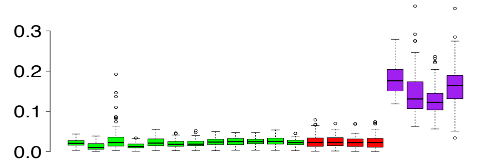	100	AP2-O targets (P=3.22e−09)	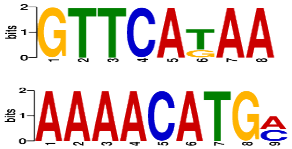 E = 8.2e-004<br>E = 1.4e-002
Cluster 41	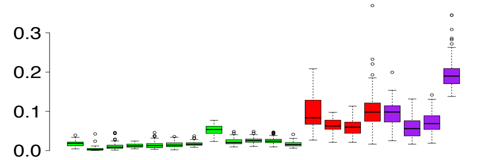	86	Translationally repressed genes (P=6.04e−11)<br>AP2-O targets (P=7.39e−10)	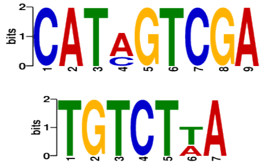 E = 2.7e-003<br>E = 3.3e-003
Cluster 42	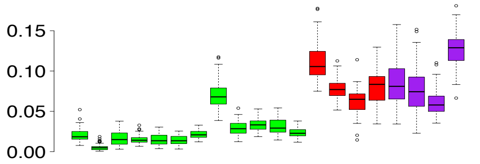	64	Female specific genes (P=5.58e−03)	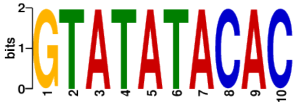 E = 2.0e-002

	Expression profile	N	Enrichment analysis (MPMP and additional datasets)(C)	Motif enriched within the cluster
Cluster 43	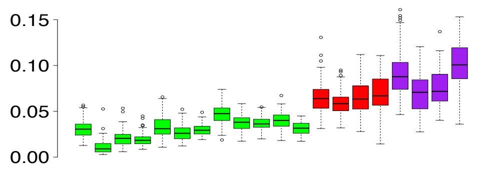	97	Male specific genes (P=2.03e−02)	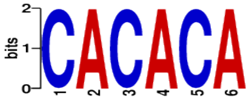 E = 5.0e-003
Cluster 44	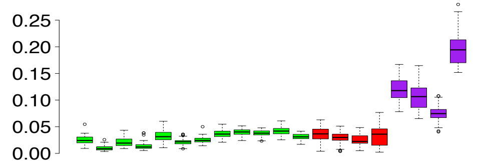	44	AP2-O targets (P=2.75e−03)	none
Cluster 45	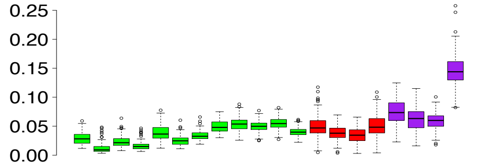	111	none	none
Cluster 46	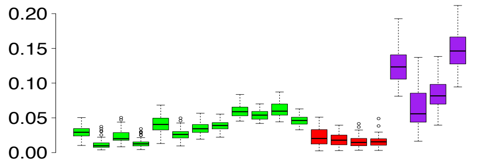	70	AP2-O targets (P=3.05e−05)	none
Cluster 47	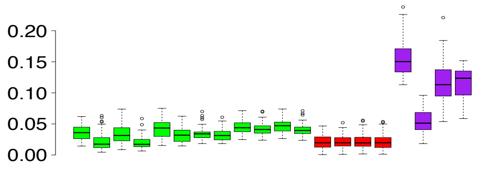	86	AP2-O targets (P=9.24e−10)	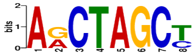 E = 2.4e-012 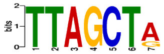 E = 3.9e-003 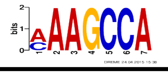 E = 3.4e-002
Cluster 48	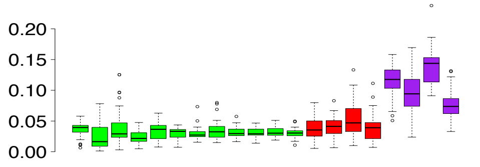	37	none	none

	Expression profile	N	Enrichment analysis (MPMP and additional datasets)(C)	Motif enriched within the cluster
Cluster 49	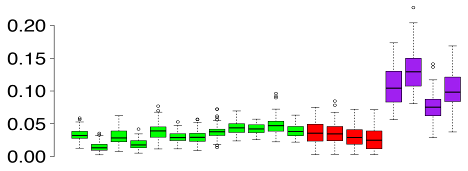	99	none	none

Data S4, related to Figure 4 and 5. Characterization of the 49 co-expression clusters generated from ApiAP2 expression data.

Expression of genes from the cluster at each strain/stage combination plotted as the average proportion of library-size-corrected reads assigned to the libraries of a given type. Schizont (green), gametocyte (red) and ookinete (purple) libraries are presented in the same order as shown in Fig. 5. N = the number of genes in a cluster. The enrichment in **Malaria Parasite Metabolic Pathways (MPMP)** (http://mpmp.huji.ac.il) and stage specific gene groups is shown if significant. P values of Fisher exact test after FDR correction are shown. GO terms enrichment for the same clusters can be found in the Data S3. Sequence motifs shown are significantly enriched within 2 kb upstream of the start codon, as compared with all the promoters in the genome. E- values of enrichment are given as calculated by the DREME software.
